# Supplementary figures and images for: Pax6 limits the competence of developing cerebral cortical cells to respond to inductive intercellular signals
Source: PLoS Biol. 2022 Sep 6;20(9):e3001563. doi: 10.1371/journal.pbio.3001563 (PMC9481180; doi:10.1371/journal.pbio.3001563)

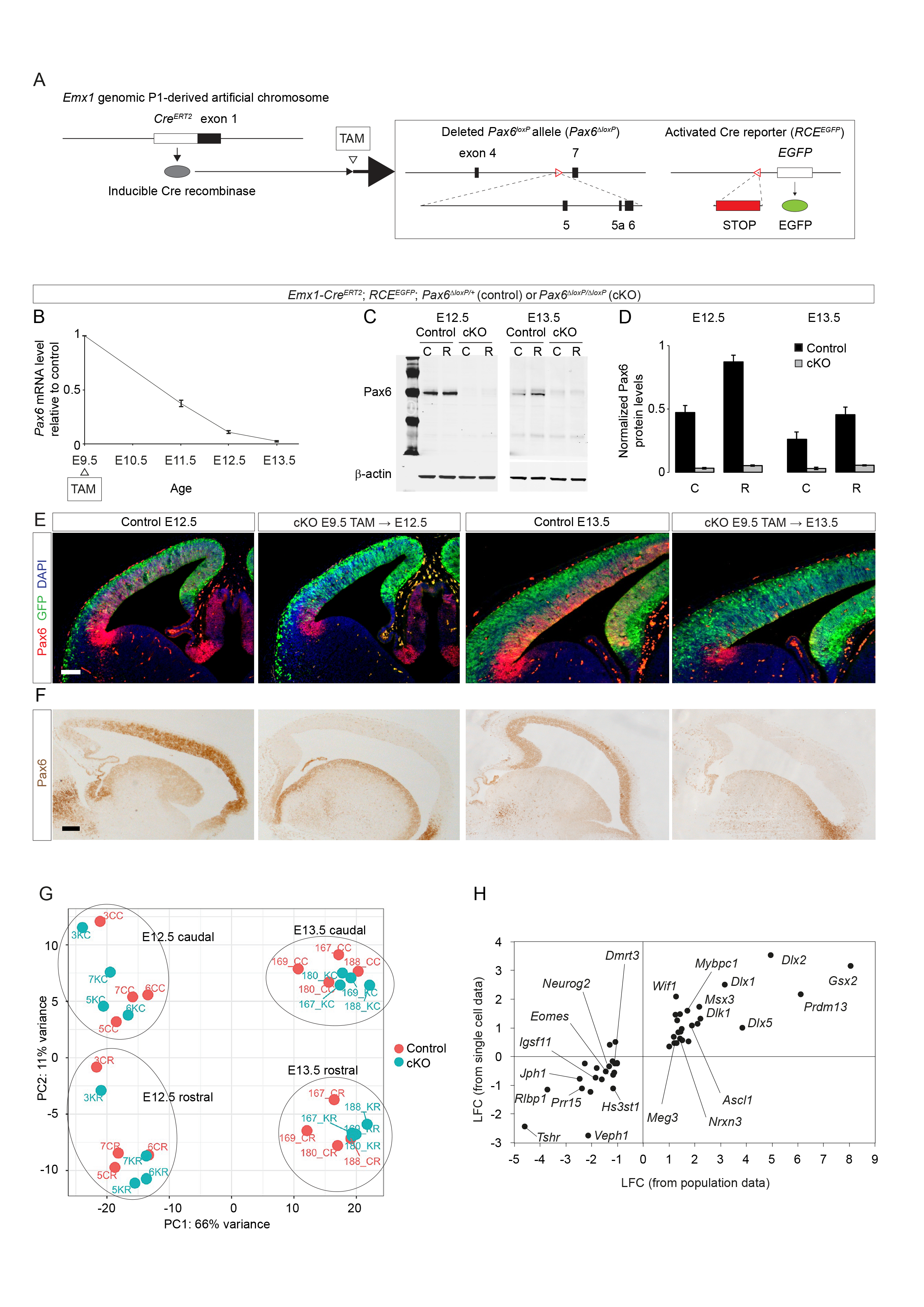

Supplement: S1 Fig — (A) Frequently used alleles: Emx1-CreERT2 producing TAM-inducible Cre recombinase [33]; Pax6loxP, from which paired domain-encoding exons were removed by Cre recombinase (Pax6ΔloxP), rendering it nonfunctional [185]; RCEEGFP, a Cre reporter producing R26R CAG-boosted EGFP [37]. Mice with deletions in both copies of Pax6 were designated conditional knockouts (Pax6 cKOs); those with a deletion in just one copy served as controls. (B) qRT-PCR measurements of Pax6 mRNA levels, normalized to those of Gapdh, after TAM administration at E9.5 were used to calculate average (±SEM) ratios between levels in Pax6 cKO and control littermates (n = 3 embryos from 3 litters at each age) (S1 Data). (C) Western blots showing Pax6 protein expression in the rostral (R) and caudal (C) cortex of control and Pax6 cKO littermates at E12.5 and E13.5 after TAM administration at E9.5. (D) Quantification of western blots at E12.5 and E13.5 after TAM administration at E9.5. Pax6 protein levels were measured relative to β-actin levels. Average levels (±SEM) were calculated (n = 3 independent repeats in each region at each age; in each case, levels in Pax6 cKOs and controls differed with p < 0.01 in Student t tests). (Note that Pax6 protein levels in control rostral cortex were almost double those in caudal control cortex at each age, in agreement with previous observations [34] (S1 Data). (E) Expression of GFP and Pax6 protein in coronal sections through the cortex of control and Pax6 cKO embryos at E12.5 and E13.5 after TAM administration at E9.5. GFP was activated by most cortical cells and Pax6 protein was lost from most cortical RGPs across almost the entire cortex, excluding a narrow ventral pallial domain where Emx1-CreERT2 was not expressed. Scale bar: 0.1 mm. (F) Expression of Pax6 protein in sagittal sections through the cortex of control and Pax6 cKO embryos at E12.5 and E13.5 after TAM administration at E9.5. Scale bar: 0.1 mm. (G) PCA on RNAseq data from CC and CR and Pax6 cKO caudal [file pbio.3001563.s001.tif]

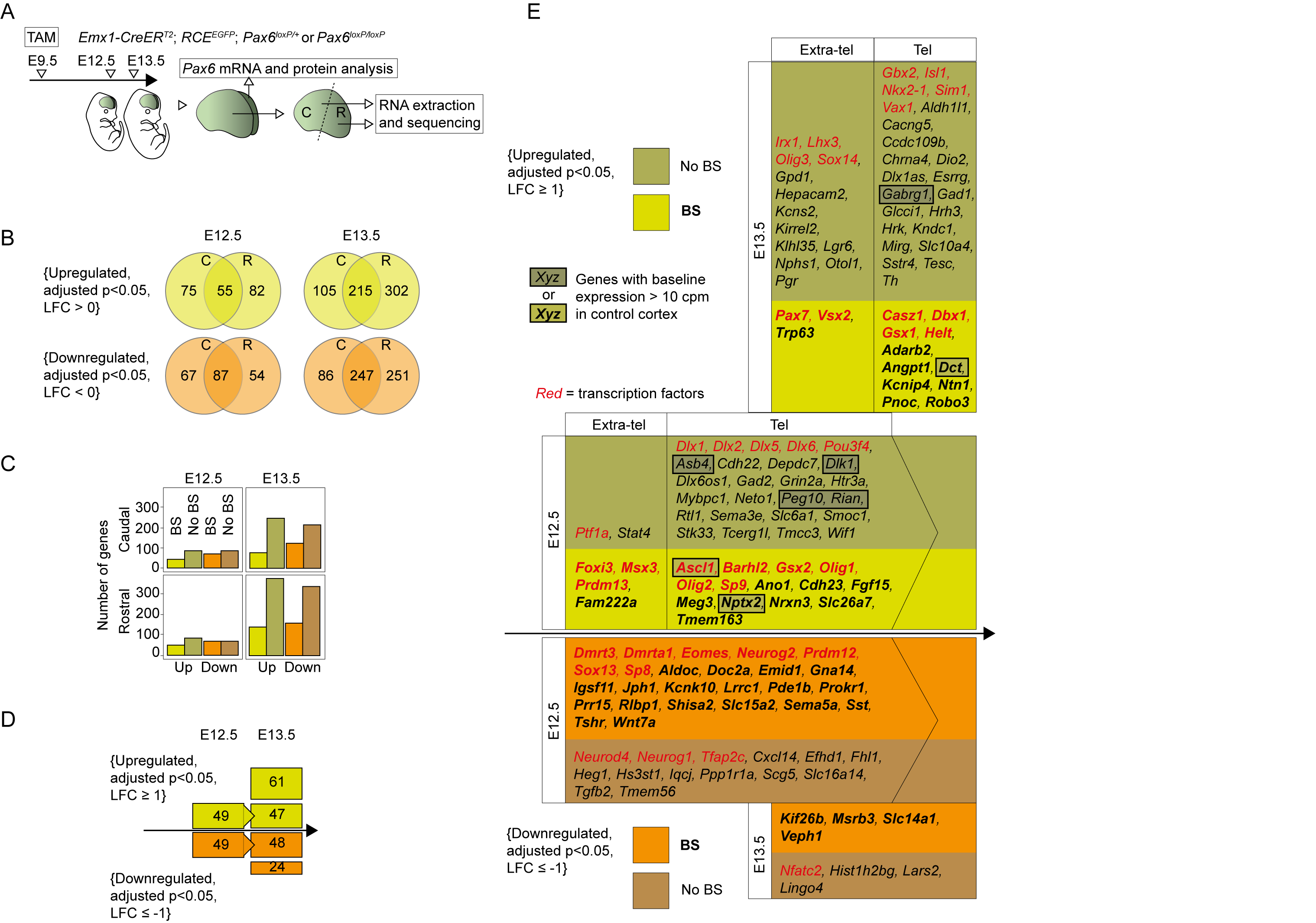

Supplement: S2 Fig — (A) The experimental procedure: TAM was administered at E9.5; one hemisphere from each E12.5 or E13.5 embryo was used to assess Pax6 mRNA and protein levels; the rostral and caudal halves of the other hemisphere were processed for RNAseq. (B) Numbers of genes with significantly up-regulated or down-regulated expression levels in caudal (C) and rostral (R) Pax6 cKO cortex (LFC). (C) Numbers of significantly up-regulated or down-regulated genes with a nearby Pax6 BS (raw RNAseq data are available at the European Nucleotide Archive accession numbers PRJEB5857 and PRJEB6774; chromatin immunoprecipitation-sequencing data are from [38]). (D) Changes with age in the numbers of significantly up-regulated or down-regulated genes that showed the largest changes in expression levels. We applied a commonly used threshold to include all functionally annotated genes (http://www.ensembl.org/index.html) that at least doubled or halved their expression levels, i.e., with an LFC in expression ≥1 (for up-regulated genes) or ≤−1 (for down-regulated genes), in at least one of the 4 combinations of age and region. This produced a subset of 183 genes: 98 genes were affected at E12.5, 95 remained so, and a further 85 were added at E13.5. To gain an initial impression of biological processes strongly associated with these genes, we passed them through the Database for Annotation, Visualization and Integrated Discovery v6.8 (DAVID v6.8; [66,67]) to obtain sets of significantly enriched GO terms (S2 Table). Some of the GO terms obtained using the up-regulated gene set described the development of cell types normally generated within the telencephalon but outside the cortex in subpallium (where cerebral cortical GABAergic interneurons and, at these ages, oligodendrocytes are made; [9,33,90]). Others described the development of nontelencephalic cell types (spinal cord, inner ear, skeletal system, and neural crest). (E) Lists of the 183 genes up-regulated and down-regulated genes that had LFCs [file pbio.3001563.s002.tif]

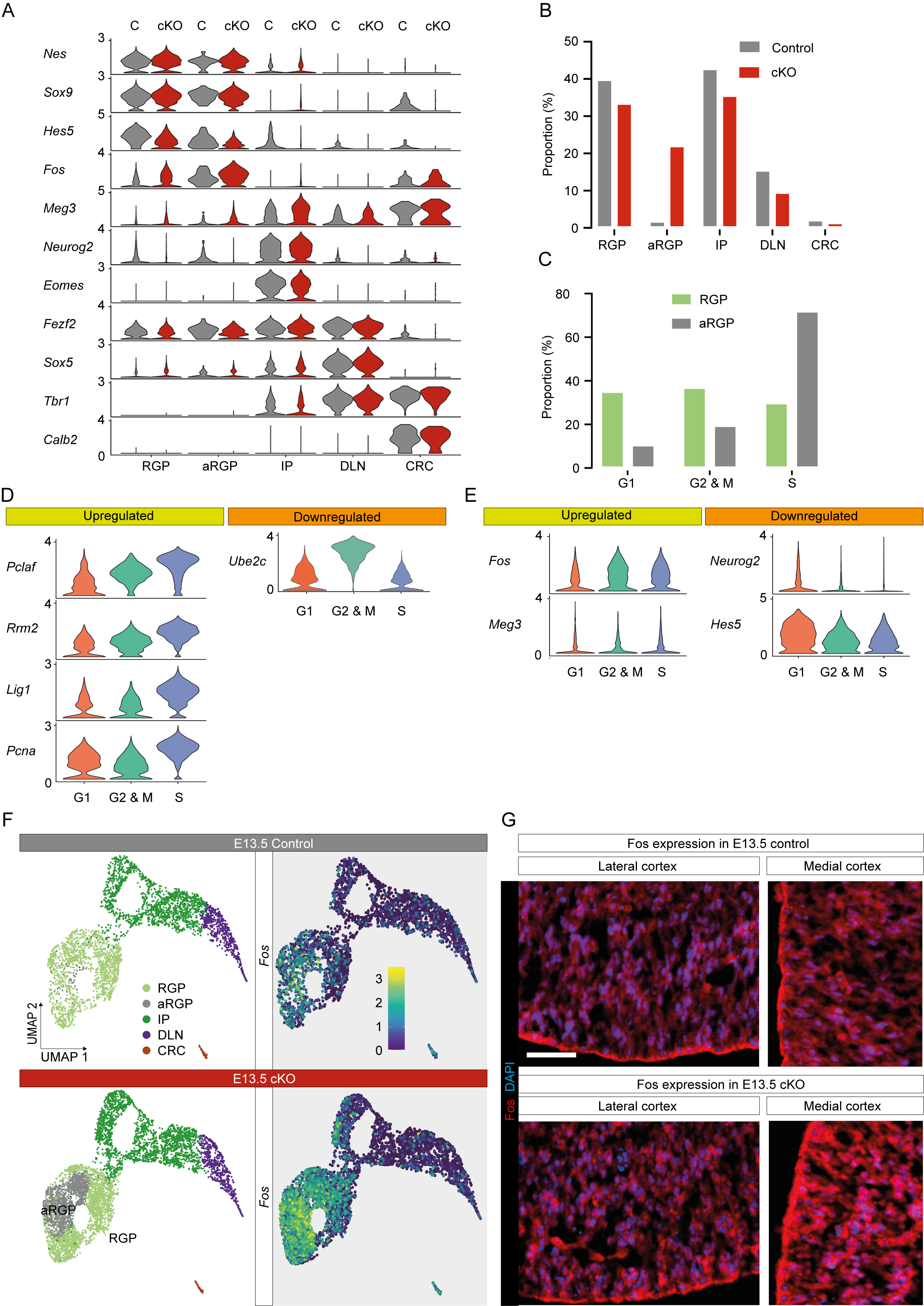

Supplement: S3 Fig — Raw data for (A-F) are available at the European Nucleotide Archive accession number PRJEB32740. (A) Violin plots of selected gene expression in each cell type from control and Pax6 cKO E13.5 cortex. (B) Proportions of cells of each type in control and Pax6 cKO E13.5 cortex. (C) The proportions of RGPs and aRGPs in different cell cycle phases in control and Pax6 cKO E13.5 cortex. We identified each RGP’s and aRGP’s cell cycle phase by profiling its expression of known cell cycle phase-selective markers [194]. We found that a relatively higher proportion of aRGPs than RGPs were in S phase. This is compatible with previous findings that Pax6 removal causes a shortening of G1, G2, and M phases [35,195]. (D, E) Violin plots of the expression levels of genes that were significantly DE between RGPs and aRGPs in different cell cycle phases in control and Pax6 cKO E13.5 cortex. The expression levels of some of the genes whose expression levels differed between aRGPs and RGPs showed large systematic variation with cell cycle phase; examples are shown in D. The aRGPs’ elevated expression of genes such as Pclaf, Rrm2, Lig1, and Pcna, whose expression levels increased in S phase, and lowered expression of genes such as Ube2c, whose expression levels increased in G2 and M phases, probably reflected the relative increase in the proportions of aRGPs in S phase. However, it was hard to explain all differences between aRGPs and RGPs in this way. For example, levels of Fos and Meg3, whose expression levels were elevated in aRGPs, and Neurog2 and Hes5, whose expression levels were lowered in aRGPs, showed much less variation with cell cycle phase (E). (F) UMAP plots of the scRNAseq data from Pax6 cKO and control cells at E13.5, reproduced from Fig 1B, showing cell types and log10-normalized expression of Fos. (G) Immunohistochemistry for Fos expression in coronal sections of rostral E13.5 lateral and medial control and Pax6 cKO cortex. Scale bar: 0.05 mm. aRGP, atypical RGP; CRC, Caja [file pbio.3001563.s003.tif]

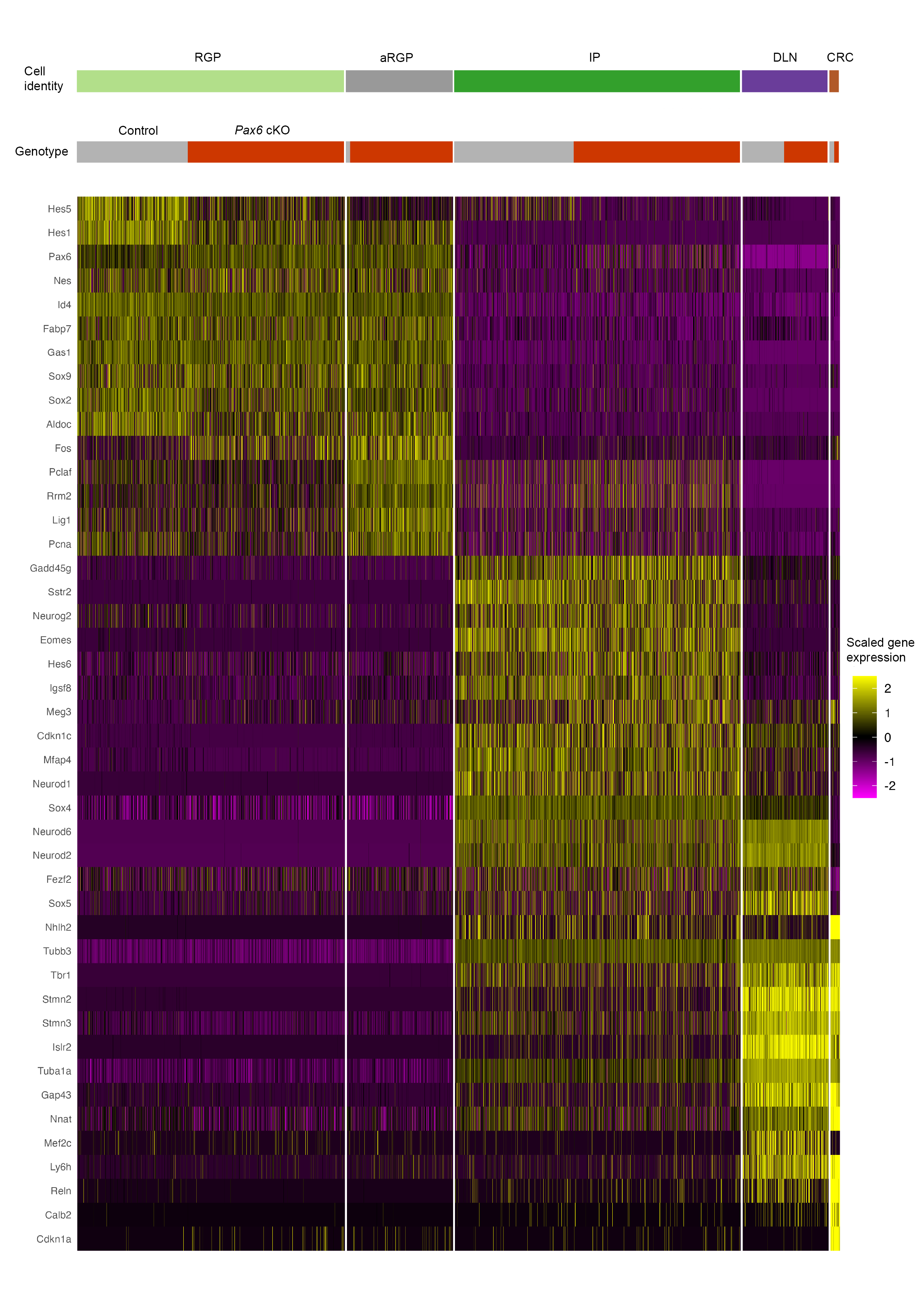

Supplement: S4 Fig — Heat map of gene expression in RGPs, aRGPs, IPs, DLNs, and CRCs in E13.5 control and Pax6 cKO cortex. Raw data are available at the European Nucleotide Archive accession number PRJEB32740. aRGP, atypical RGP; CRC, Cajal–Retzius cell; DLN, deep layer neuron; IP, intermediate progenitor; Pax6 cKO, Pax6 conditional knockout; RGP, radial glial progenitor. (TIF) [file pbio.3001563.s004.tif]

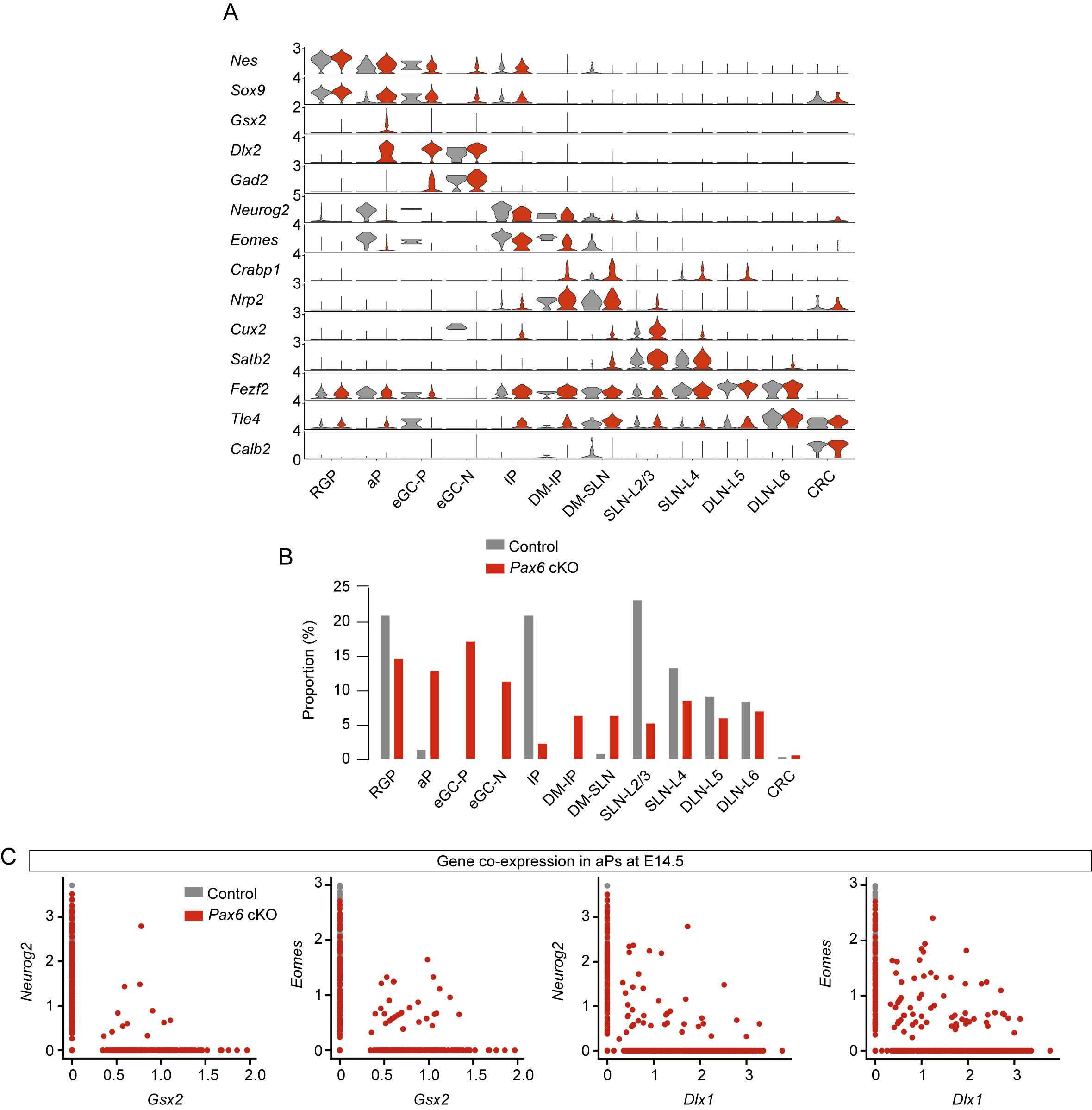

Supplement: S5 Fig — Raw data are available at the European Nucleotide Archive accession number PRJEB27937. (A) Violin plots of selected gene expression in each cell type from control and Pax6 cKO E14.5 cortex. (B) Proportions of cells of each type in control and Pax6 cKO E14.5 cortex. (C) Graphs showing coexpression of markers of normal cortical cells (Neurog2 and Eomes) and normal subcortical cells (Gsx2 and Dlx1) in some aPs in E14.5 Pax6 cKOs. aP, atypical progenitor; CRC, Cajal–Retzius cell; DLN-L5 and DLN-L6, layer 5 or layer 6 deep layer neurons; DM-IP and DM-SLN, intermediate progenitor or superficial layer neuron in dorsomedial cortex; eGC-P and eCG-N, proliferating or nonproliferating ectopic GABAergic cells; IP, intermediate progenitor; Pax6 cKO, Pax6 conditional knockout; RGP, radial glial progenitor; SLN-L2/3 and SLN-L4, layer 2/3 or layer 4 superficial layer neurons. (TIF) [file pbio.3001563.s005.tif]

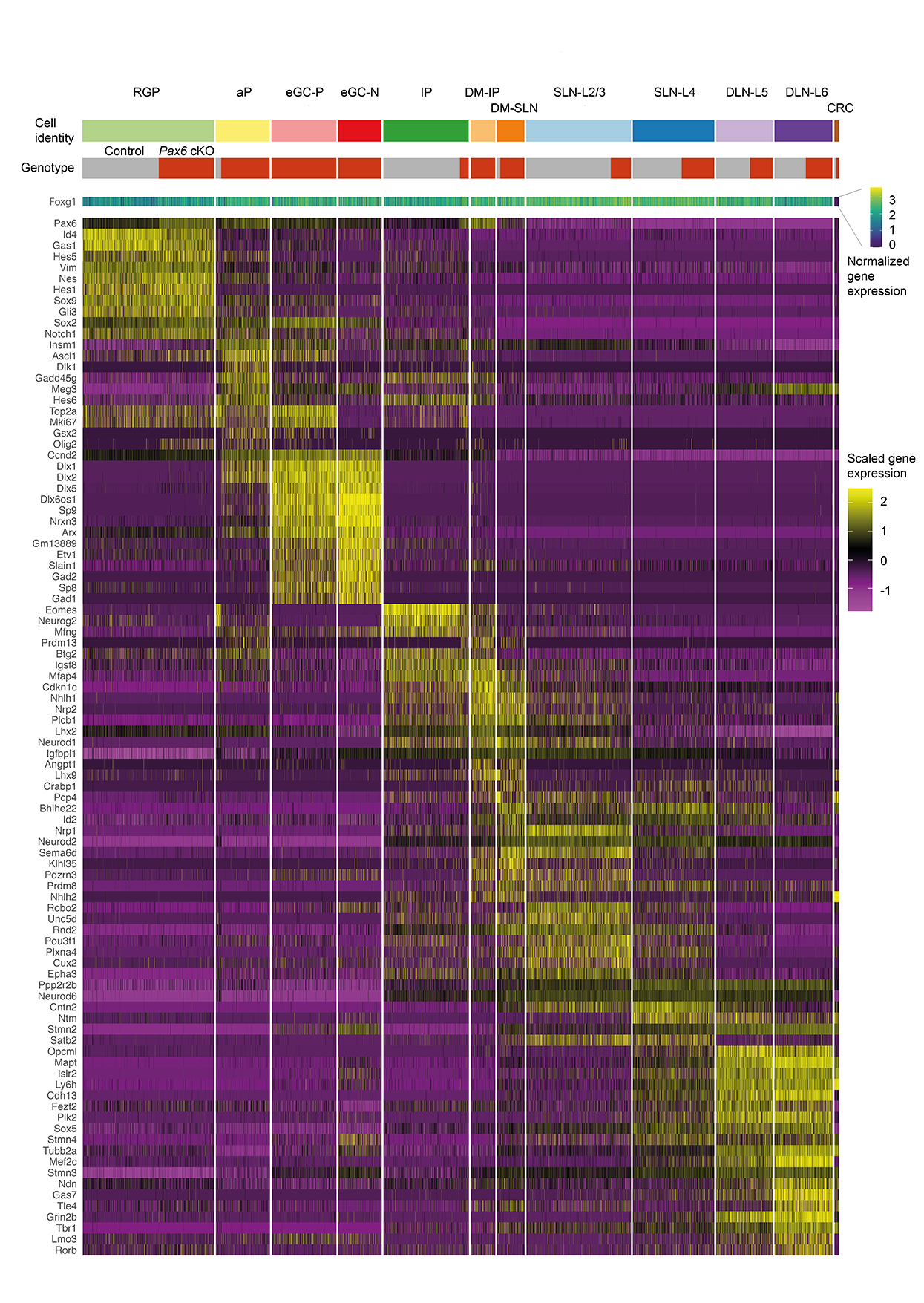

Supplement: S6 Fig — Heat map of gene expression in RGPs, aPs, eGC-Ps and eCG-Ns, IPs, DM-IPs and DM-SLNs, SLN-L2/3s and SLN-L4s, DLN-L5s and DLN-L6s, and CRCs in E14.5 control and Pax6 cKO cortex. Note the increased levels of Pax6 mRNA in Pax6 cKO cells, which was anticipated because the loss of Pax6 protein would have removed the negative feedback constraining the transcription of residual Pax6 coding sequence (Manuel and colleagues, 2007). Raw data are available at the European Nucleotide Archive accession number PRJEB27937. aP, atypical progenitor; CRC, Cajal–Retzius cell; DLN-L5 and DLN-L6, layer 5 or layer 6 deep layer neurons; DM-IP and DM-SLN, intermediate progenitor or superficial layer neuron in dorsomedial cortex; eGC-P and eCG-N, proliferating or nonproliferating ectopic GABAergic cells; IP, intermediate progenitor; Pax6 cKO, Pax6 conditional knockout; RGP, radial glial progenitor; SLN-L2/3 and SLN-L4, layer 2/3 or layer 4 superficial layer neurons. (TIF) [file pbio.3001563.s006.tif]

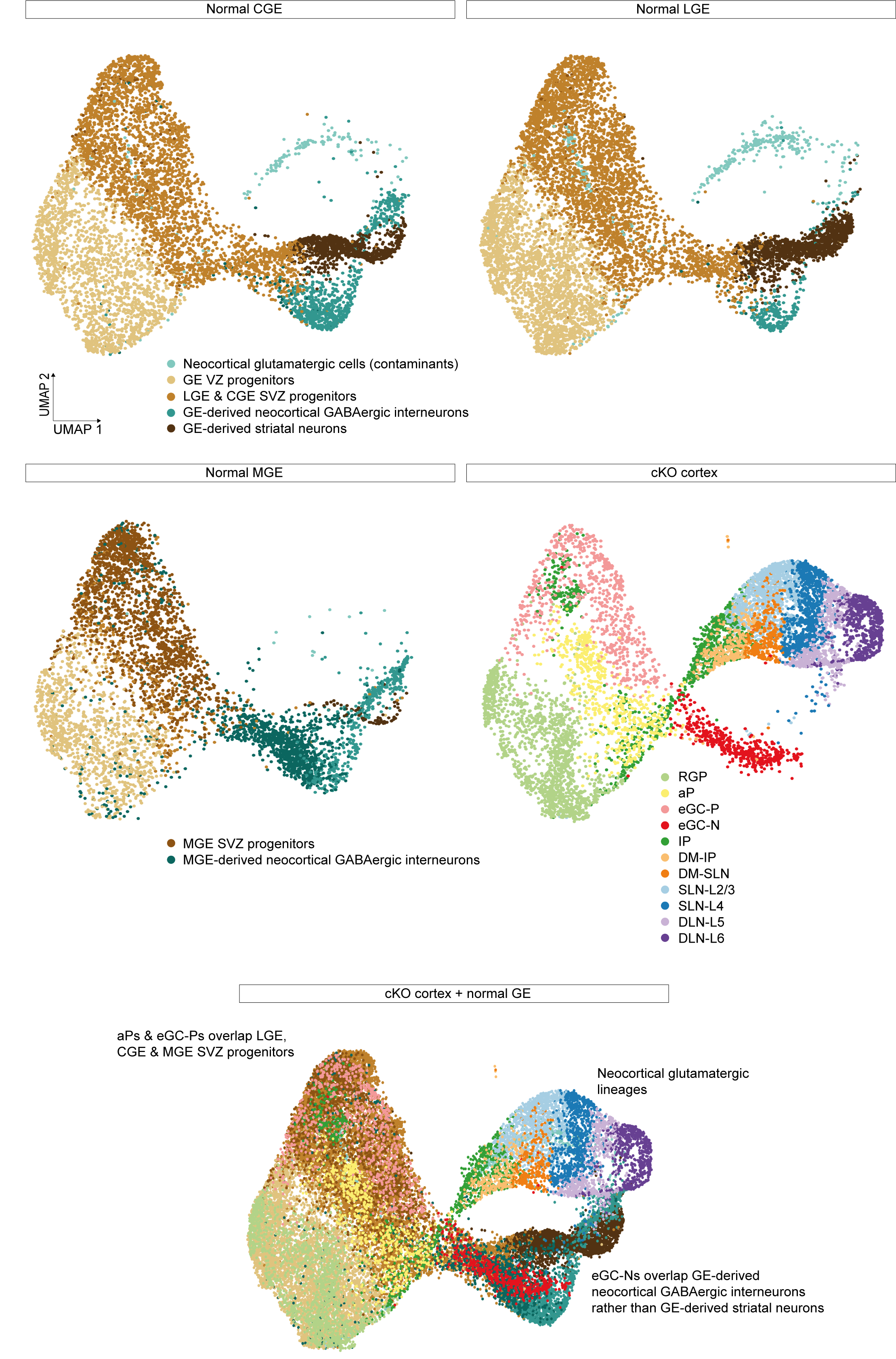

Supplement: S7 Fig — UMAP plots of scRNAseq data from normal E14.5 ventral telencephalon [86] integrated with our scRNAseq data from E14.5 Pax6 cKO cortex (raw data are available at the European Nucleotide Archive accession number PRJEB27937). Abbreviations for cortex in S5A Fig. CGE, caudal ganglionic eminence; GE, ganglionic eminence; LGE, lateral ganglionic eminence; MGE, medial ganglionic eminence; Pax6 cKO, Pax6 conditional knockout; SVZ, subventricular zone; UMAP, uniform manifold approximation and projection; VZ, ventricular zone. (TIF) [file pbio.3001563.s007.tif]

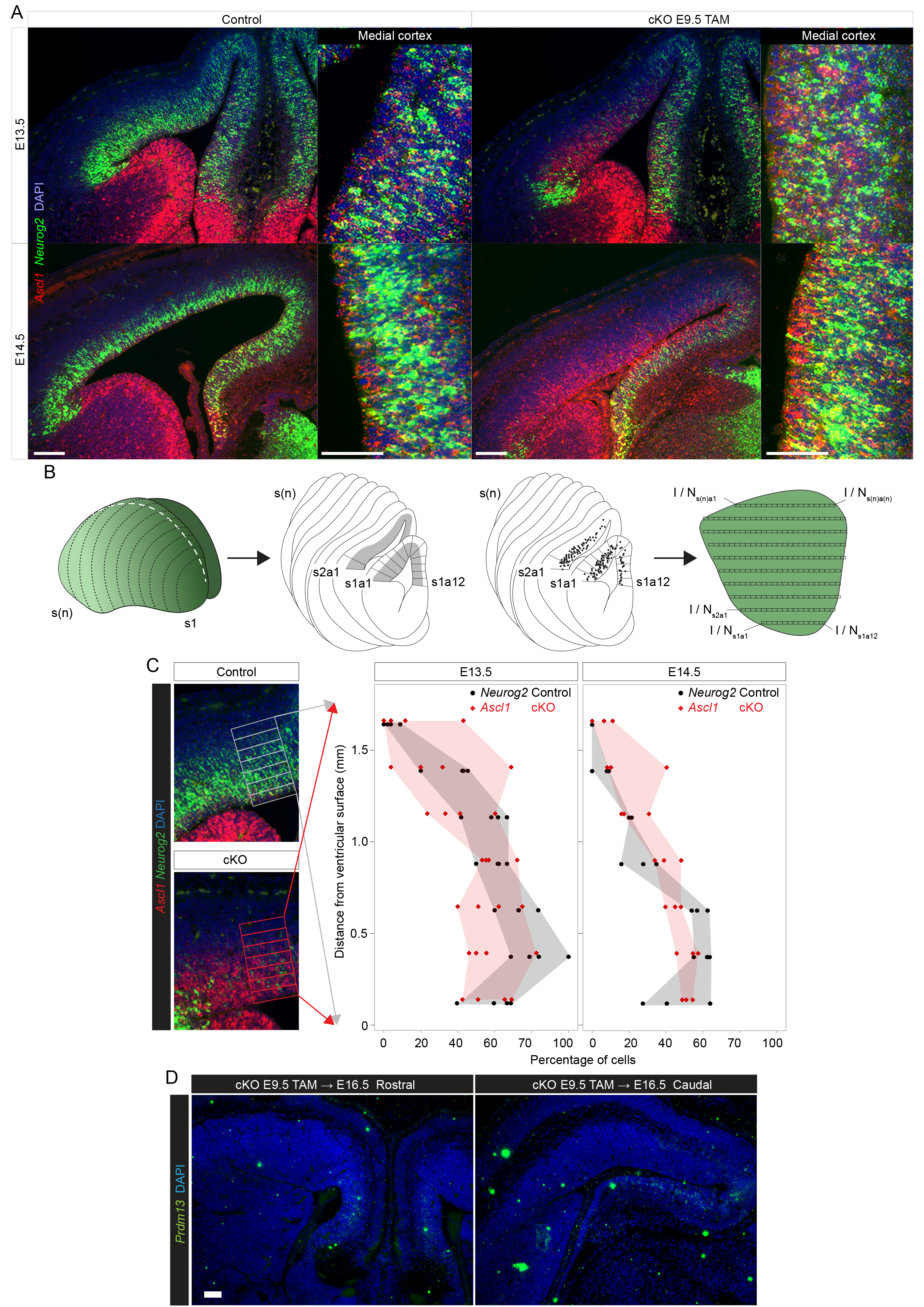

Supplement: S8 Fig — (A) In situ hybridizations for Ascl1 and Neurog2 in control and Pax6 cKO cortex at E13.5 and E14.5. Scale bars: 0.1 mm. (B) Method for obtaining a surface-view reconstruction of labeling across the cortex from a series of equally spaced coronal sections (s1 to s(n)). Relative intensity of label (I) or labeled cell number (N) were measured in areas (a1 to a(n)) with average width (through cortical depth) of 0.1 mm in each section. Values were then laid out on a flattened representation of the cortical surface and maps generated by interpolation. (C) Quantification showing the similarity in the distributions with depth of the proportions of cells expressing Neurog2 mRNA in control lateral cortex and Ascl1 mRNA in Pax6 cKO lateral cortex at E13.5 and E14.5. Data were obtained using 25 μm × 100 μm bins, as shown, from the lateral cortex in centrally located coronal sections through the brains of 4 Pax6 cKOs and 4 controls at E13.5 and 3 Pax6 cKOs and 3 controls at E13.5. Data points are for individual animals and shaded areas show the range of values with depth (S2 Data). (D) In situ hybridization for Prdm13 in rostral and caudal Pax6 cKO cortex at E16.5 after tamoxifen administration at E9.5. Scale bar: 0.1 mm. Pax6 cKO, Pax6 conditional knockout. (TIF) [file pbio.3001563.s008.tif]

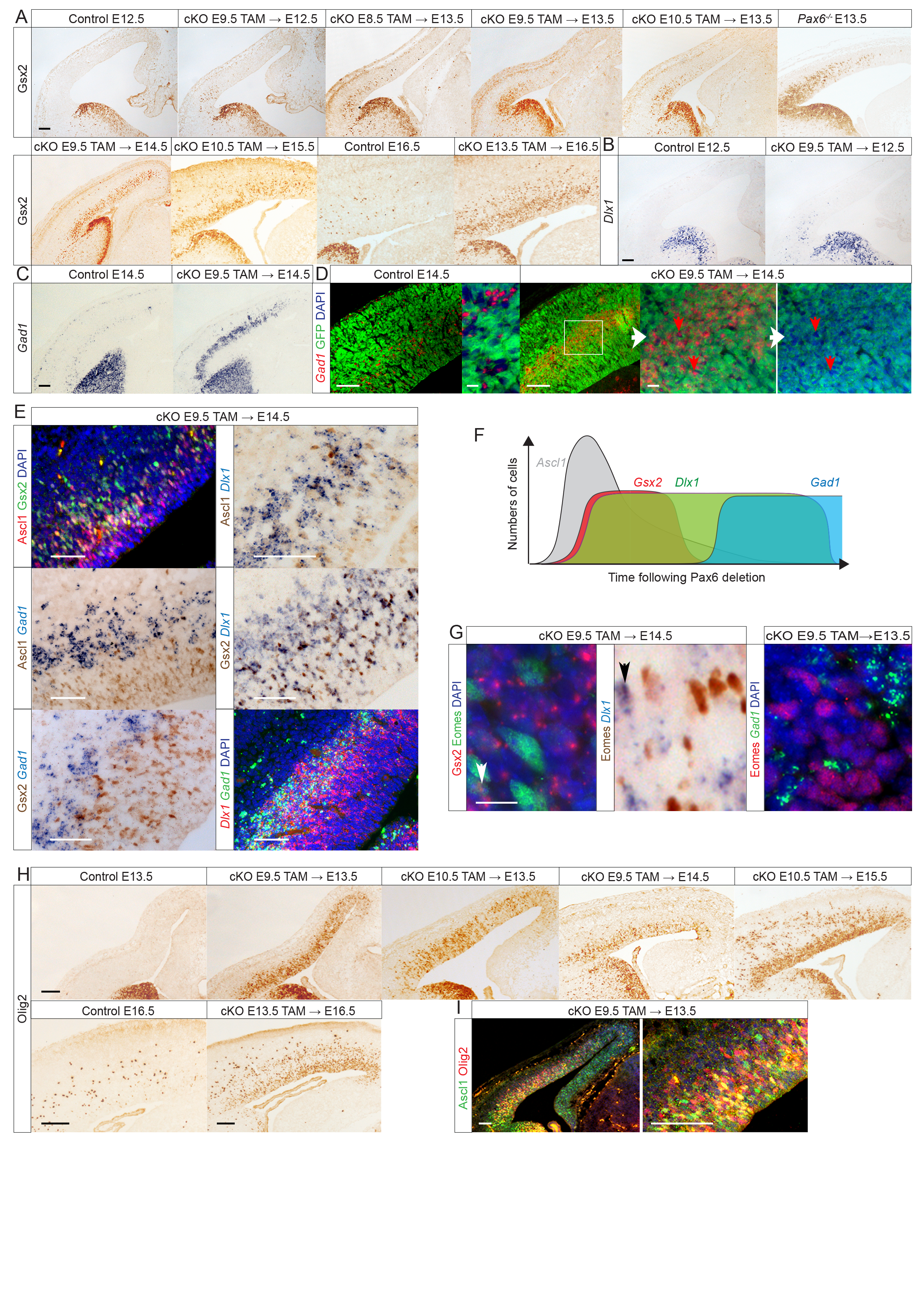

Supplement: S9 Fig — (A) Immunoreactivity for Gsx2 in control and Pax6 cKO cortex between E12.5 and E16.5 following tamoxifen at E8.5, E9.5, E10.5, or E13.5 and in the cortex of E13.5 constitutive Pax6−/− embryos. Scale bar: 0.1 mm. (B, C) In situ hybridizations for Dlx1 and Gad1 in control and Pax6 cKO cortex at E12.5 and E14.5 following tamoxifen at E9.5. Scale bars: 0.1 mm. (D) In situ hybridizations for Gad1 and immunoreactivity for GFP in control and Pax6 cKO cortex at E14.5 following tamoxifen at E9.5, showing enlargement of boxed area with and without Gad1 staining. Red arrows in Pax6 cKO cortex: examples of Gad1+ cells that were GFP-negative. Scale bars: 0.1 mm and 0.01 mm. (E) Fluorescence and colorimetric immunoreactivity for Ascl1 and Gsx2 and in situ hybridizations for Dlx1 and Gad1 in Pax6 cKO lateral cortex at E14.5 following tamoxifen at E9.5. Scale bars: 0.1 mm. (F) Summary of gene expression changes in (F) with time following Pax6 deletion. (G) Fluorescence and colorimetric immunoreactivity for Gsx2 and Eomes and in situ hybridizations for Dlx1 and Gad1 in Pax6 cKO cortex at E13.5 and E14.5 following tamoxifen at E9.5. Scale bar: 0.01 mm. (H, I) Colorimetric and fluorescence immunoreactivity for Olig2 and Ascl1 in control and Pax6 cKO cortex between E13.5 and E16.5 following tamoxifen at E9.5, E10.5 or E13.5. Scale bars: 0.1 mm. GFP, green fluorescent protein; Pax6 cKO, Pax6 conditional knockout. (TIF) [file pbio.3001563.s009.tif]

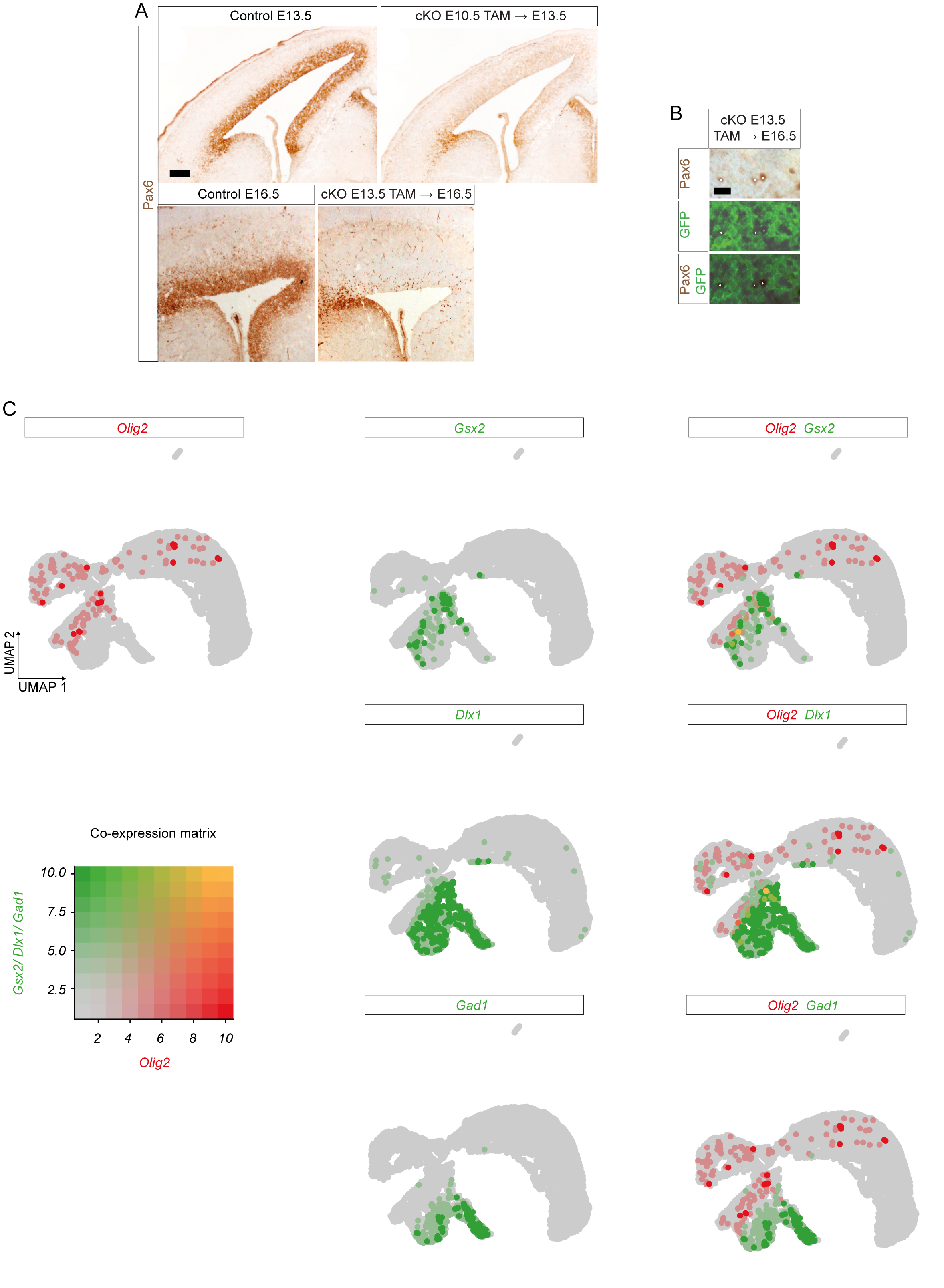

Supplement: S10 Fig — (A) Expression of Pax6 protein in coronal sections through the cortex of control and Pax6 cKO embryos at E13.5 after tamoxifen administration at E10.5 and at E16.5 after tamoxifen administration at E13.5. Scale bar: 0.1 mm. (B) Expression of Pax6 protein and GFP in sections through the Pax6 cKO cortex at E16.5 after tamoxifen administration at E13.5. The vast majority of cortical RGPs were Pax6-negative. Many of the few remaining Pax6+ cells were GFP-negative (examples marked with white dots). Scale bar: 0.01 mm. (C) UMAP plots from Pax6 cKO cortex at E14.5 showing relative expression and coexpression levels of Olig2, Gsx2, Dlx1, and Gad1 in each cell (on a 0–10 scale, 10 was the highest expression level of the gene in question). Raw data are available at the European Nucleotide Archive accession number PRJEB27937. GFP, green fluorescent protein; Pax6 cKO, Pax6 conditional knockout; RGP, radial glial progenitor; UMAP, uniform manifold approximation and projection. (TIF) [file pbio.3001563.s010.tif]

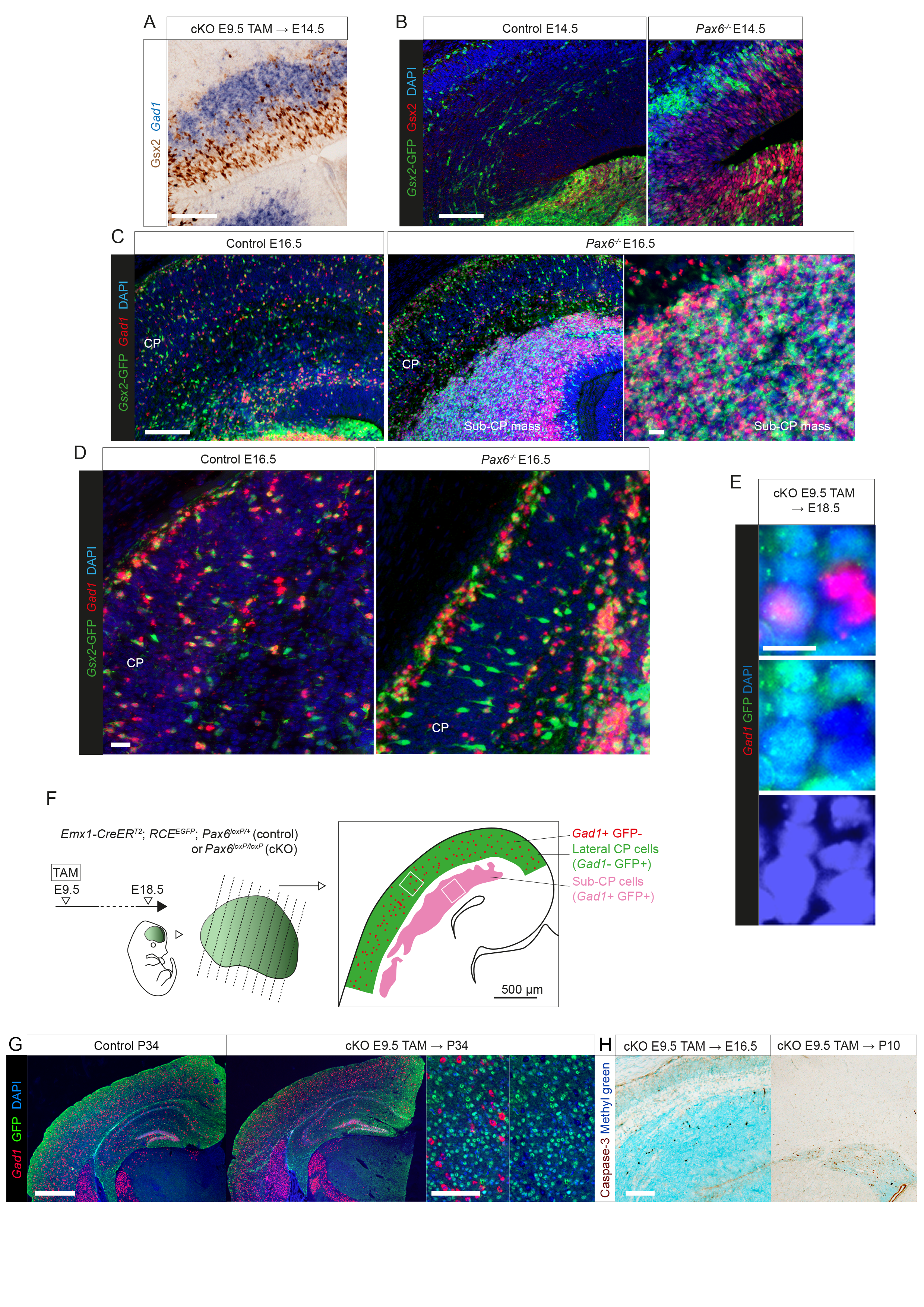

Supplement: S11 Fig — (A) Colorimetric immunoreactivity for Gsx2 and in situ hybridization for Gad1 in E14.5 Pax6 cKO lateral cortex after tamoxifenE9.5. Scale bar: 0.1 mm. (B) Fluorescence immunoreactivity for Gsx2 and GFP, the latter marking Gsx2-lineage cells, in E14.5 control and Pax6−/− telencephalon. Scale bar: 0.1 mm. (C, D) Fluorescence immunoreactivity for GFP+ (Gsx2-lineage) and in situ hybridization for Gad1+ cells in E16.5 control and Pax6−/− lateral cortex. Scale bars: 0.1 mm and 0.01 mm. (E) Three Gad1+ cells in the CP of an E18.5 Pax6 cKO (tamoxifenE9.5) carrying the RCEEGFP Cre-reporter. One was Emx1-lineage (GFP+). Scale bar: 0.01 mm. (F) Experimental design for analysis of the effects of Pax6 deletion on cortical cell numbers (Fig 4H). The Emx1-CreERT2 allele with tamoxifenE9.5 was used to delete Pax6; embryos carried a GFP reporter of Cre activity; embryos were collected at E18.5; regularly spaced coronal sections were double-stained for GFP protein and Gad1 mRNA. We measured the surface area of the CP of the lateral cortex and the surface area of the sub-CP masses of one hemisphere in each section and interpolated to estimate the volumes of these structures in each embryo. We used random sampling (e.g., white boxes) to measure the average densities of cells in each region. We then calculated their total numbers in each region in each embryo. Data from 4 littermate pairs from separate mothers were used to produce Fig 4H. (G) Fluorescence immunoreactivity for GFP (Emx1-lineage) and in situ hybridization for Gad1+ cells in P34 control and Pax6 cKO cortex after tamoxifen at E9.5. High-magnification images: same region of CP with and without Gad1 staining. Scale bar: 1 mm and 0.1 mm. (H) Immunoreactivity for Caspase-3 in CP and in sub-CP masses in Pax6 cKO cortex at E16.5 and at P10 after tamoxifen at E9.5. Scale bar: 0.1 mm. CP, cortical plate; eGC, ectopic GABAergic cell; GFP, green fluorescent protein; Pax6 cKO, Pax6 conditional knockout. (TIF) [file pbio.3001563.s011.tif]

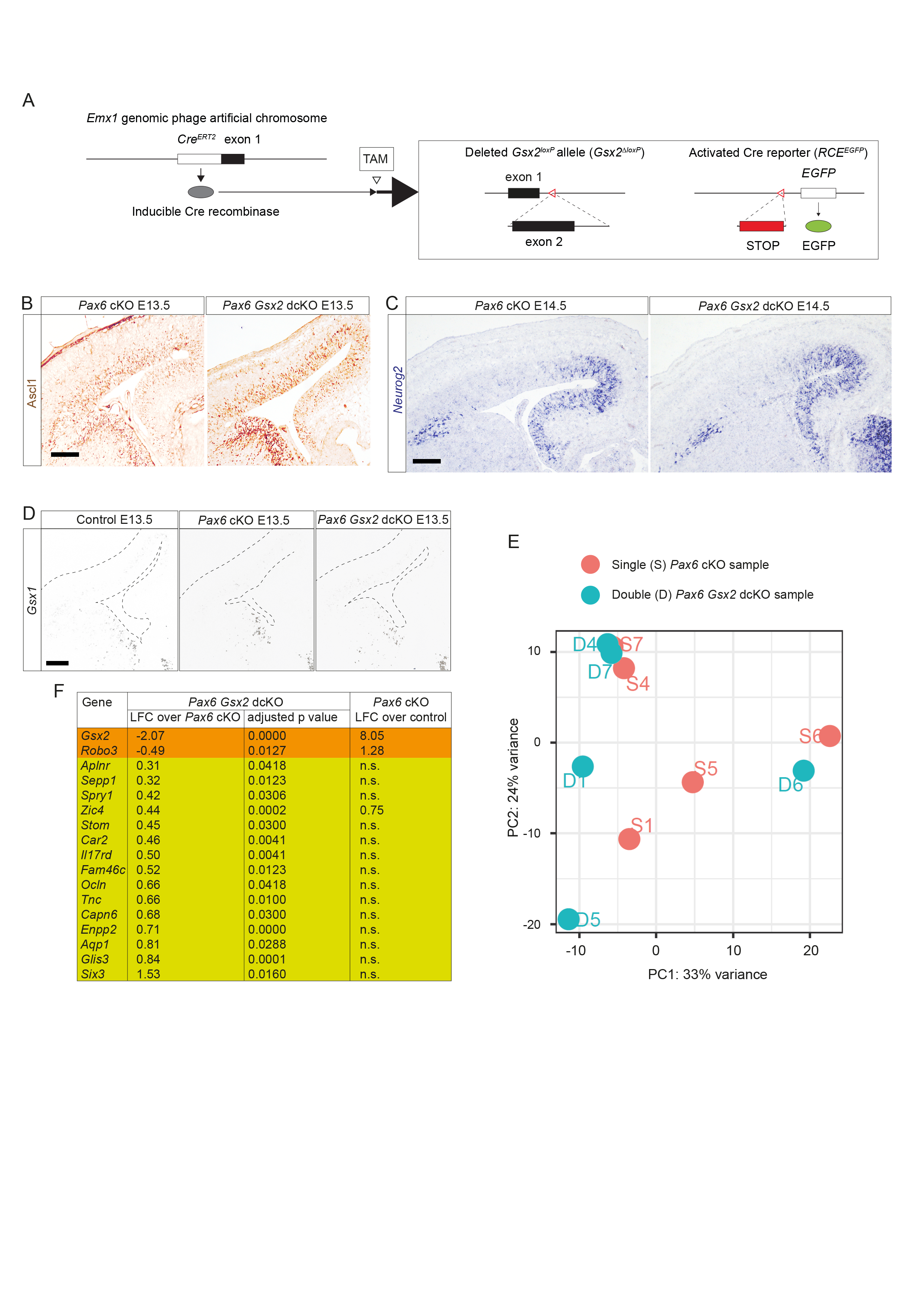

Supplement: S12 Fig — (A) Alleles used to delete conditionally Gsx2: Emx1-CreERT2 producing TAM-inducible Cre recombinase [33]; Gsx2loxP [47]; RCEEGFP [37]. (B, C) Immunoreactivity for Ascl1 and in situ hybridization for Neurog2 in Pax6 cKO and Pax6 Gsx2 dcKO cortex at E13.5 and E14.5. Scale bar: 0.1 mm. (D) In situ hybridization for Gsx1 in control, Pax6 cKO and Pax6 Gsx2 dcKO cortex at E13.5. Scale bar: 0.1 mm. (E) PCA on RNAseq data from Pax6 cKO (n = 5 embryos) and Pax6 Gsx2 dcKO (n = 5 embryos) cortex at E13.5. Data were from the rostral half of the cortex, where the proportion of cells activating Gsx2 after Pax6 deletion was highest. Raw data are available at the European Nucleotide Archive accession number PRJEB21105. (F) A list of all genes that were significantly down-regulated or up-regulated (adjusted p < 0.05) in Pax6 Gsx2 dcKO cortex compared to Pax6 cKO cortex and their LFCs in Pax6 cKO compared to control cortex (n.s., not significant). Raw data are available at the European Nucleotide Archive accession numbers PRJEB21105 and PRJEB5857. dcKO, double conditional KO; LFC, log2 fold change; Pax6 cKO, Pax6 conditional knockout; PCA, principal component analysis; TAM, tamoxifen. (TIF) [file pbio.3001563.s012.tif]

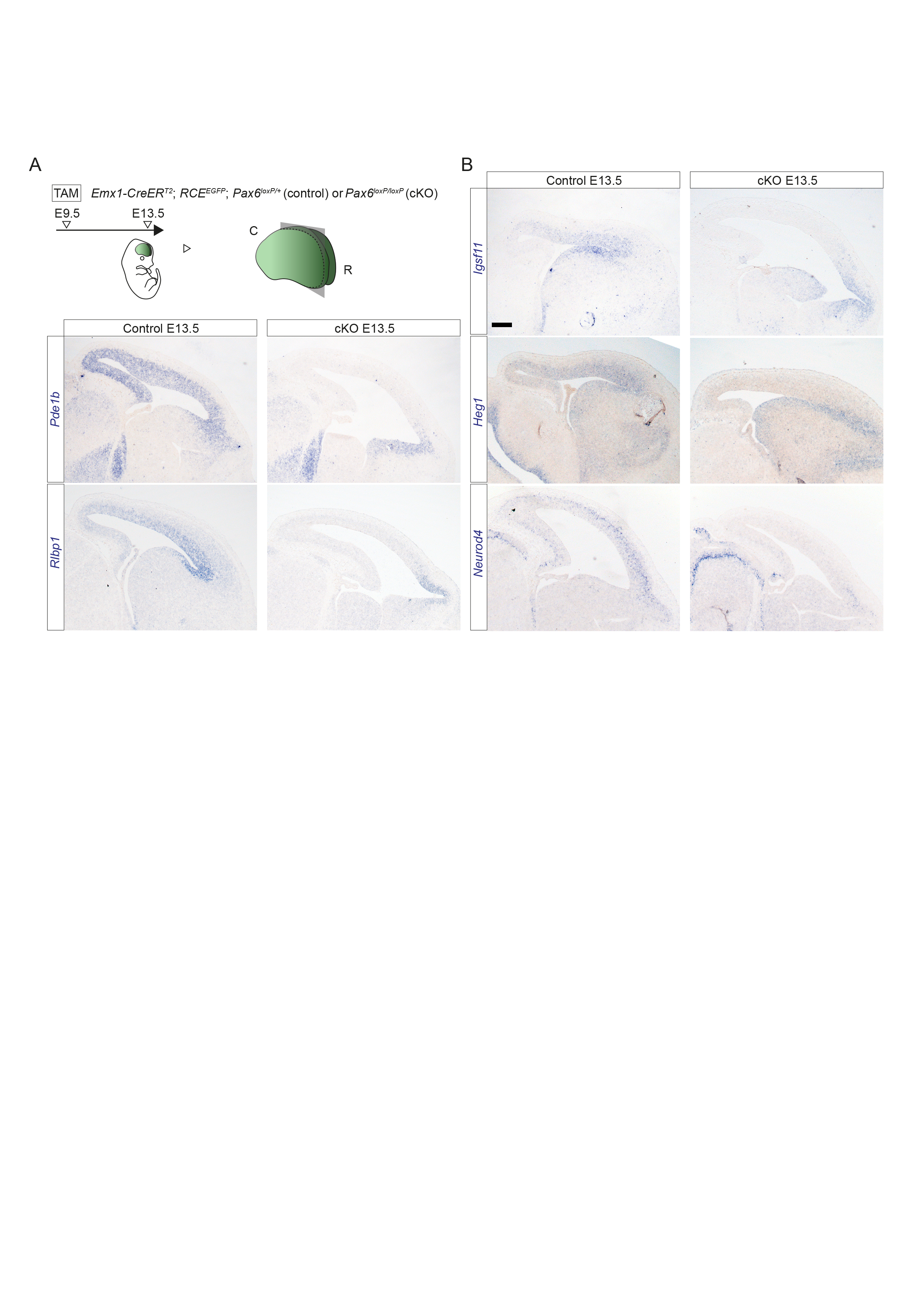

Supplement: S13 Fig — (A) TAM was administered at E9.5 to generate control and Pax6 cKO embryos; brains were sectioned sagittally at E13.5. (B) In situ hybridizations for Pde1b, Rlbp1, Igsf11, Heg1, and Neurod4 in E13.5 control and Pax6 cKO cortex. Scale bar: 0.1 mm. Pax6 cKO, Pax6 conditional knockout; TAM, tamoxifen. (TIF) [file pbio.3001563.s013.tif]

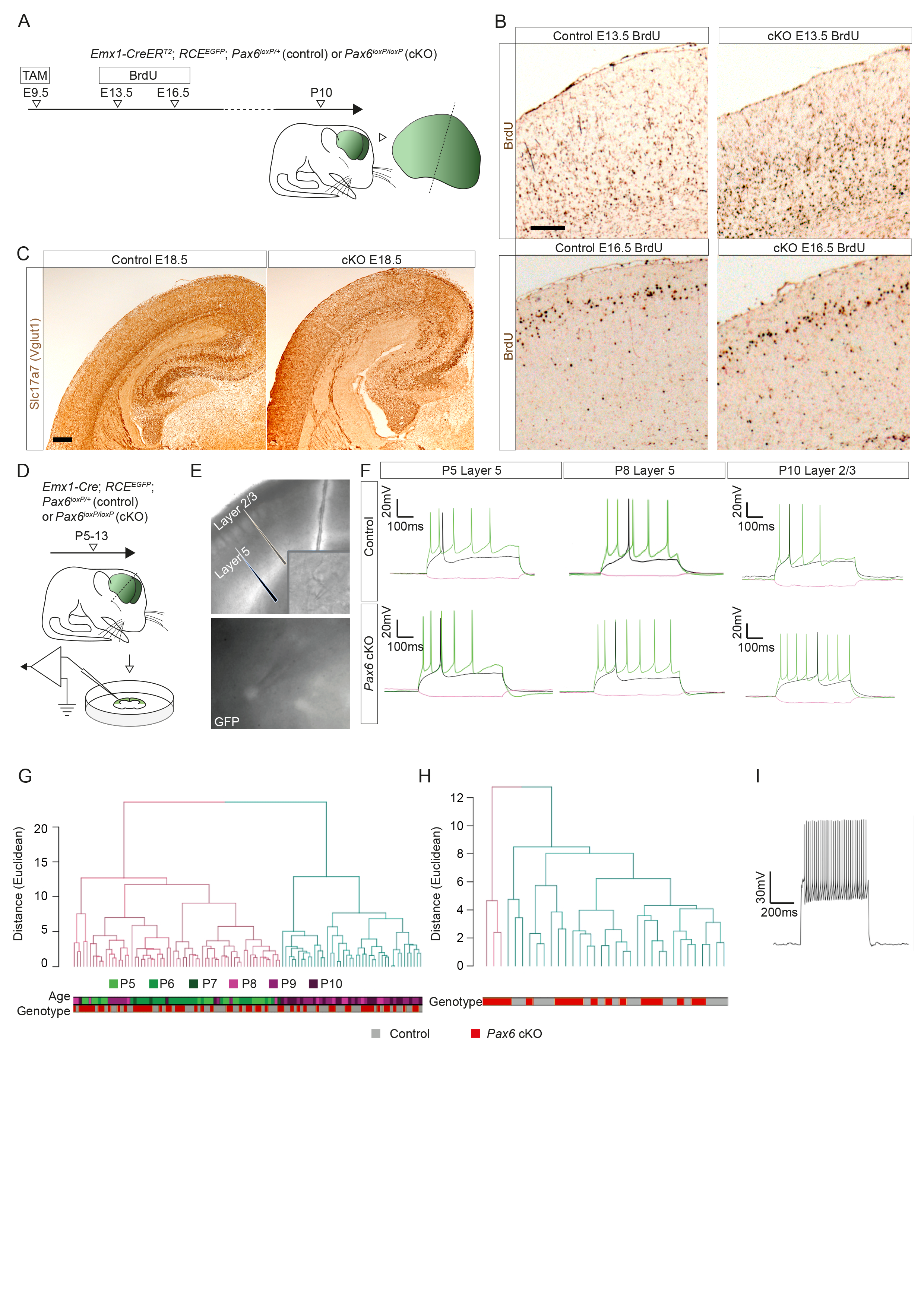

Supplement: S14 Fig — (A) The experimental procedure for (B): TAM was administered at E9.5 to generate control and Pax6 cKO embryos; BrdU was injected on either E13.5 or E16.5; brains were sectioned coronally and analysed at P10. (B) BrdU-labeled cells in P10 lateral CP; experimental procedure in (A). Scale bar: 0.1 mm. (C) Immunoreactivity for Slc17a7 (formerly Vglut1) in E18.5 control and Pax6 cKO cortex after TAM at E9.5. Scale bar: 0.1 mm. (D) The experimental procedure for (E-I): slices from P5–13 Emx1-Cre; RCEEGFP; Pax6loxP/+ (control) or Pax6loxP/loxP (Pax6 cKO) mice were used for electrophysiology. (E) GFP+ cells in layers 2/3 and 5 of somatosensory cortex area 1 (S1) were targeted for whole-cell current clamp recordings (electrodes are visible in inset in upper panel and targeting a GFP+ cell in lower panel). (F) Examples of membrane voltage responses to progressive current injections for control and Pax6cKO cortex (500 ms square steps; hyperpolarizing step: −25 pA; depolarizing steps: rheobase and double rheobase). (G) Unsupervised hierarchical clustering analysis for S1 layer 5. Features used for clustering are listed in S5 Table. Purple tones represent older pups (P8-P10). Green tones represent younger pups (P5-P7). Genotype bar indicates control cells (gray; n = 54) and Pax6cKO cells (red; n = 55). Cells from both genotypes were spread across the clusters with no segregation of Pax6cKO cells. Silhouette coefficient analysis suggested the optimal number of clusters was 2 (silhouette coefficient = 0.26, k = 2), which separated cells mainly by age (Sheet C in S5 Data). (H) Unsupervised hierarchical clustering analysis for S1 layers 2/3. Features used for clustering are listed in S5 Table. Silhouette coefficient analysis suggested the optimal number of clusters was 2 (silhouette coefficient = 0.44, k = 2). Clustering split the cells into 2 main branches, with one containing 3 cells all from Pax6cKO mice, but this separation might have occurred by chance (Barnard unconditional tw [file pbio.3001563.s014.tif]

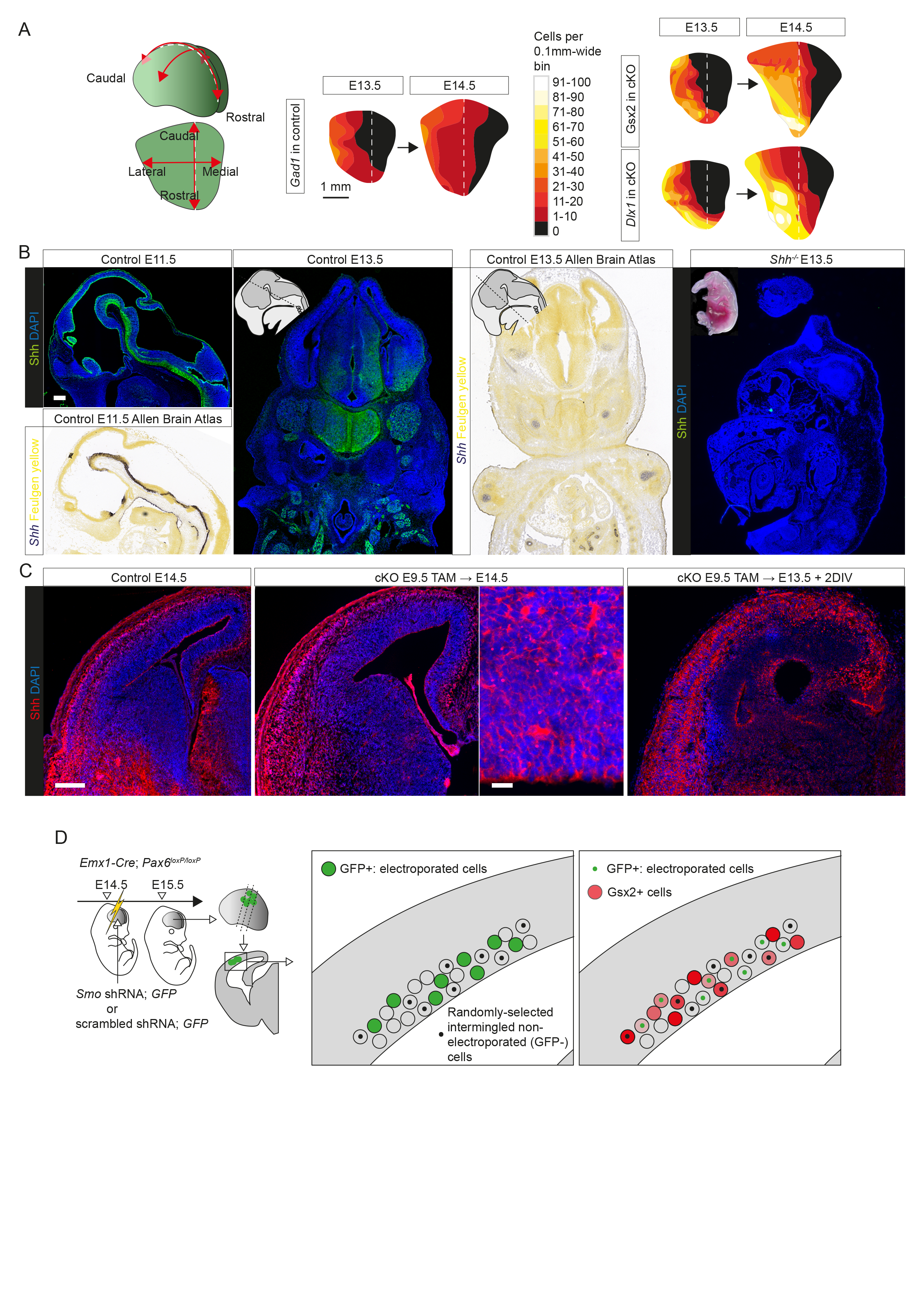

Supplement: S15 Fig — (A) Flattened surface views of the cortex showing the densities of Gad1+ cells at E13.5 and E14.5 in controls: Gad1+ cells spread in increasing numbers across the cortex from lateral to medial with a similar spatiotemporal pattern to the spread of Gsx2 and Dlx1 activation after TAM at E9.5 (maps for Gsx2 and Dlx1 reproduced from Fig 3H). (B) Validation of the Shh antibody: comparison of staining patterns, in both neural and nonneural tissues [196], with in situ hybridization patterns from the Allen Brain Atlas in E11.5 and E13.5 controls and lack of staining in E13.5 Shh−/− mutants (kindly provided by Laura Lettice and Bob Hill, Edinburgh University). Scale bar: 0.1 mm. (C) Immunoreactivity for Shh in control telencephalon at E14.5 and in Pax6 cKO telencephalon after TAM at E9.5 at E14.5 and after 2 DIV from E13.5 (Fig 6A). Scale bars: 0.1 mm and 0.01 mm. (D) Following the experimental procedure reproduced from Fig 6K: constructs expressing Smo shRNA + GFP or scrambled shRNA + GFP were electroporated into the cortex of E14.5 Pax6 cKO embryos made using Emx1-Cre; electroporated cells were analysed at E15.5. Blind to Gsx2 expression, we identified 80–100 GFP+ cells and a random selection of 80–100 intermingled GFP− cells in each of 3 embryos from 3 litters given Smo shRNA and each of 4 embryos from 3 litters given scrambled shRNA. The intensity of Gsx2 immunoreactivity was then measured in all of these cells and frequency distributions of intensities in electroporated versus non-electroporated cells were compared in brains that received Smo shRNA and in brains that received scrambled shRNA (results in Fig 6M). DIV, day in vitro; eGC, ectopic GABAergic cell; GFP, green fluorescent protein; Pax6 cKO, Pax6 conditional knockout; TAM, tamoxifen. (TIF) [file pbio.3001563.s015.tif]

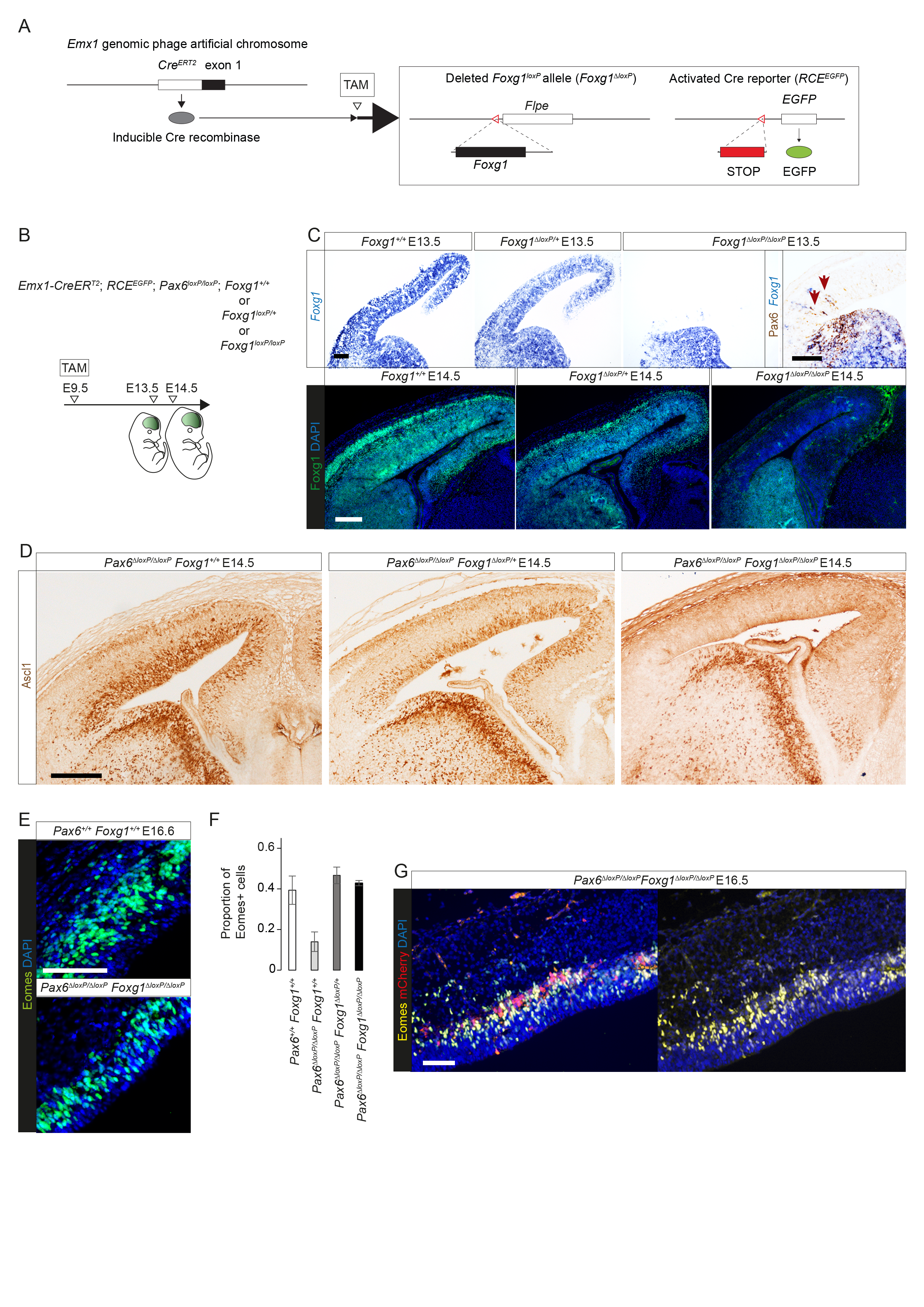

Supplement: S16 Fig — (A) Deletion of Foxg1, here with Emx1-CreERT2 and TAM, removes its coding region [184] and activates the Cre reporter. (B) TAM was administered at E9.5 to generate Pax6 cKOs in which both, one, or neither Foxg1 allele(s) were also deleted; brains were analysed at E13.5 and E14.5. The Pax6loxP allele was shown in S1A Fig. (C) In situ hybridizations for Foxg1 and immunohistochemistry for Foxg1 and Pax6 following deletion of both, one or neither Foxg1 allele(s) at E13.5 and E14.5. Arrows: A few cells remained undeleted and formed small clones expressing both Foxg1 and Pax6. Scale bars: 0.1 mm. (D) Immunoreactivity for Ascl1 in E14.5 cortex from Pax6 cKO embryos in which neither, one or both Foxg1 allele(s) were deleted by TAM at E9.5. Scale bars: 0.1 mm. (E) Immunoreactivity for Eomes in E16.5 lateral cortex from control embryos and Pax6 Foxg1 double KOs. Scale bar: 0.1 mm. (F) Proportions of cells in the ventricular and subventricular zones of E16.5 lateral cortex expressing Eomes in control embryos and embryos of the 3 genotypes in (B) (averages ± SD of n = 3; Pax6 single cKO average was significantly lower than all others, p < 0.05 in all comparisons; Student t tests) (Sheet D in S7 Data). (G) Results of experiment in Fig 7F: coexpression of Eomes and mCherry in a coronal section. Scale bar: 0.1 mm. Pax6 cKO, Pax6 conditional knockout; TAM, tamoxifen. (TIF) [file pbio.3001563.s016.tif]

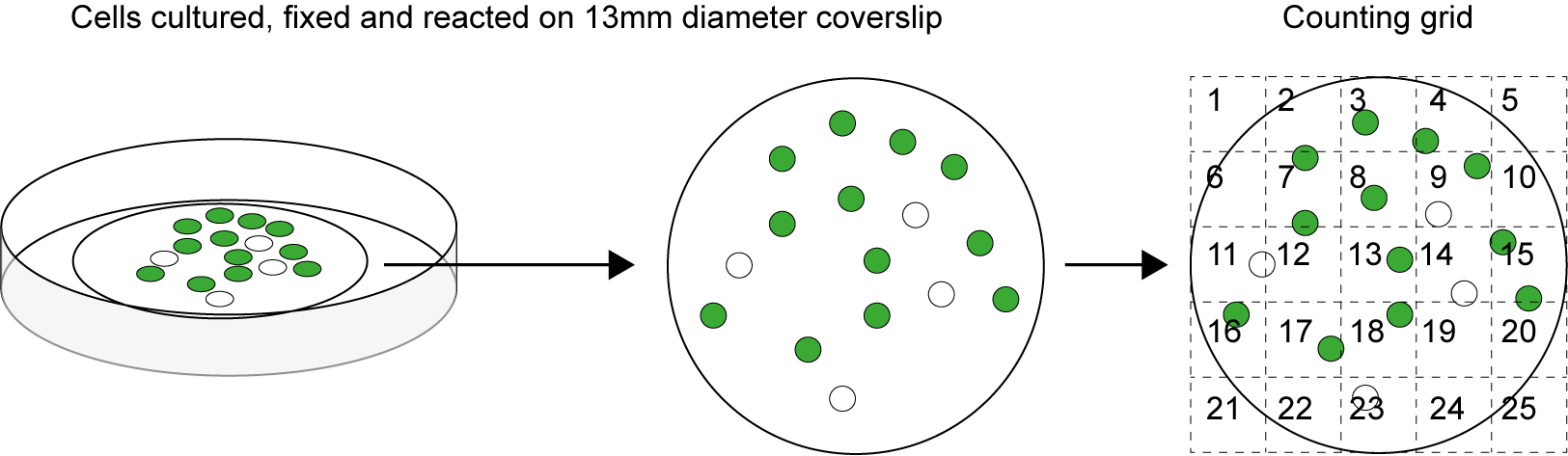

Supplement: S17 Fig — Method for quantification of the effects of SAG on numbers of GFP+ cells expressing various markers: Cells were cultured on coverslips and, after fixation and reaction, counting grids were used to sample 5 randomly selected areas from each coverslip. Several independent biological repeats were used for each condition (i.e., each concentration of SAG or vehicle alone, on Pax6 cKO or control cells) (Fig 8). GFP, green fluorescent protein; Pax6 cKO, Pax6 conditional knockout; SAG, signaling agonist. (TIF) [file pbio.3001563.s017.tif]

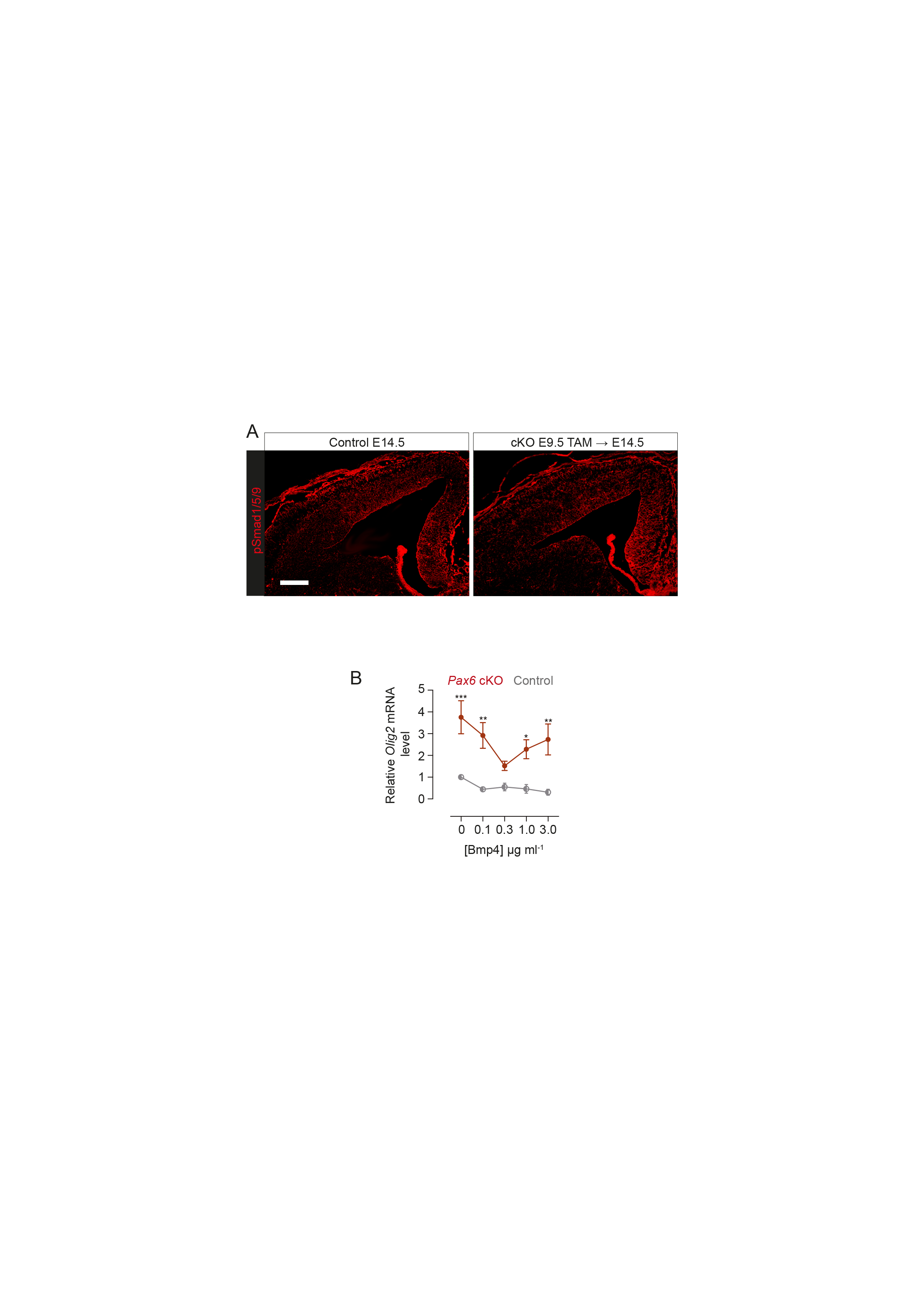

Supplement: S18 Fig — (A) Immunoreactivity for phospho-Smad1/5/9 in E14.5 control and Pax6 cKO cortex after tamoxifenE9.5. Scale bar: 0.1 mm. (B) Concentration-response measured using qRT-PCR: Olig2 levels (averages ± SEM; values are relative to the average level in control cortex treated with 0 Bmp4) in control and Pax6 cKO slices with increasing concentrations of Bmp4 (n = 3 independent cultures at each concentration). Two-way ANOVA showed significant effects of genotype on Olig2 (p < 0.005), but no significant effect of Bmp4 concentration and no significant interaction effect. Differences between genotypes at each Bmp4 concentration were tested with Bonferroni’s method for comparison of means (*, p < 0.05; **, p < 0.01; ***, p < 0.005) (S9 Data). Pax6 cKO, Pax6 conditional knockout; qRT-PCR, quantitative real-time polymerase chain reaction. (TIF) [file pbio.3001563.s018.tif]

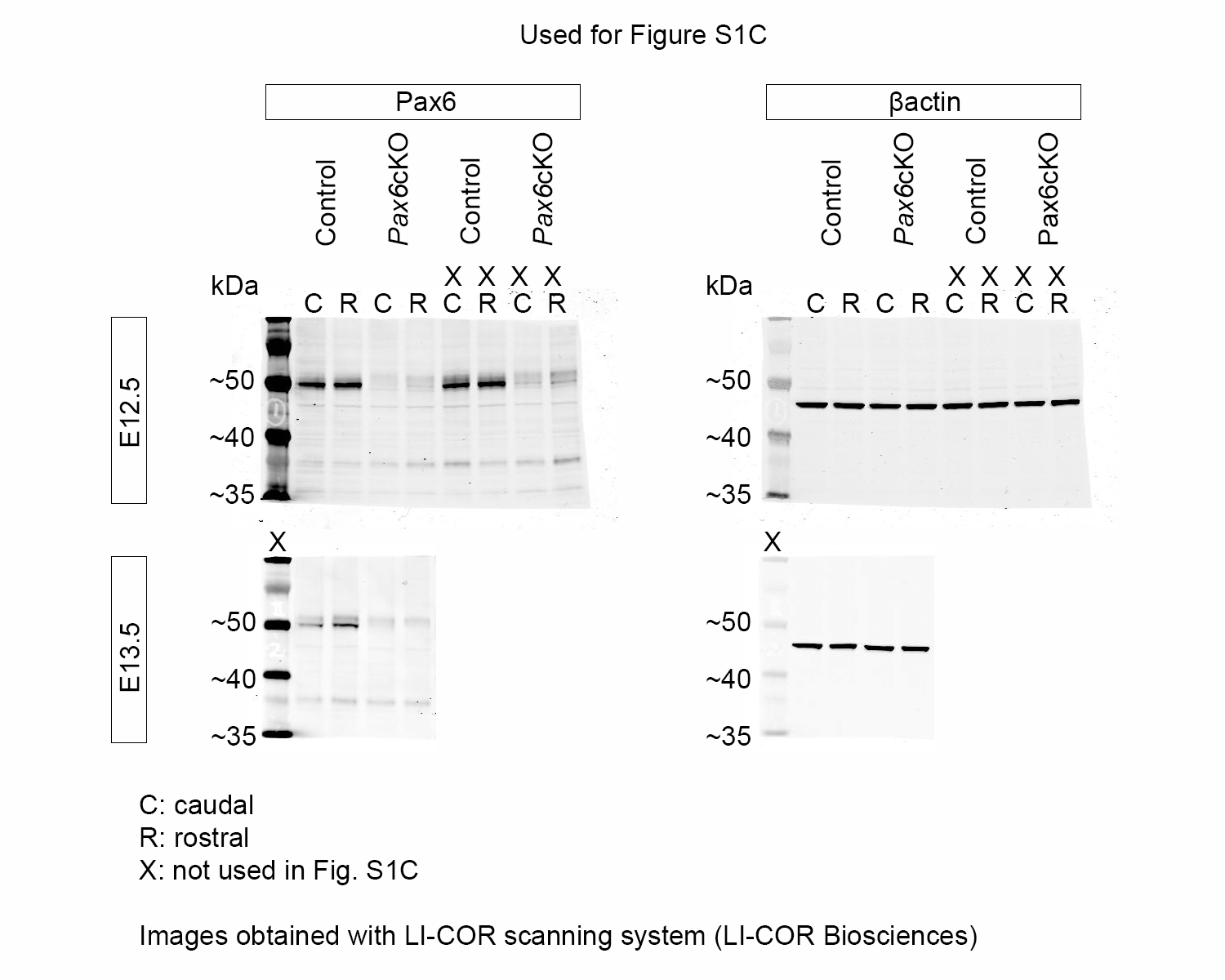

Supplement: S1 Raw Images — (TIF) [file pbio.3001563.s033.tif]
