# Supplementary material for: Pax6 limits the competence of developing cerebral cortical cells to respond to inductive intercellular signals
Source: PLoS Biol. 2022 Sep 6;20(9):e3001563. doi: 10.1371/journal.pbio.3001563 (PMC9481180; doi:10.1371/journal.pbio.3001563)
Supplement: S3 Table — (DOCX) [file pbio.3001563.s021.docx]

|  | **Upregulated Gene** | **References** |
| --- | --- | --- |
|  |  |  |
| **Telencephalic** | *Adarb2* | [1] |
|  | *Aldh1l1* | [2] |
|  | *Angpt1* | [3] |
|  | *Ano1* | [4] |
|  | *Asb4* | [5] |
|  | *Ascl1* | [5-7] |
|  | *Barhl2* | [8-10] |
|  | *Cacng5* | [11] |
|  | *Casz1* | [12,13] |
|  | *Ccdc109b* | [3] |
|  | *Cdh22* | [11,14,15] |
|  | *Cdh23* | [16,17] |
|  | *Chrna4* | [18] |
|  | *Dbx1* | [19,20] |
|  | *Dct* | [3,21] |
|  | *Depdc7* | [3] |
|  | *Dio2* | [10,22] |
|  | *Dlk1* | [23,24] |
|  | *Dlx1* | [5,7] |
|  | *Dlx1as* | [5] |
|  | *Dlx2* | [5,7] |
|  | *Dlx5* | [5] |
|  | *Dlx6* | [5] |
|  | *Dlx6os1* | [25] |
|  | *Esrrg* | [26] |
|  | *Fgf15* | [27] |
|  | *Gabrg1* | [17] |
|  | *Gad1* | [5,7] |
|  | *Gad2* | [28] |
|  | *Gbx2* | [5,29,30] |
|  | *Glcci1* | [31] |
|  | *Grin2a* | [17] |
|  | *Gsx1* | [5,7] |
|  | *Gsx2* | [5,7,32-37] |
|  | *Helt* | [7,38,39] |
|  | *Hrh3* | [40] |
|  | *Hrk* | [41] |
|  | *Htr3a* | [42] |
|  | *Isl1* | [7,43,44] |
|  | *Kcnip4* | [11] |
|  | *Kndc1* | [17] |
|  | *Meg3* | [24] |
|  | *Mirg* | [24] |
|  | *Mybpc1* | [17] |
|  | *Neto1* | [45] |
|  | *Nkx2-1* | [1,7,46] |
|  | *Nptx2* | [47] |
|  | *Nrxn3* | [48,49] |
|  | *Ntn1* | [50-52] |
|  | *Olig1* | [53] |
|  | *Olig2* | [7,54] |
|  | *Peg10* | [55] |
|  | *Pnoc* | [11,56] |
|  | *Pou3f4* | [57] |
|  | *Rian* | [24] |
|  | *Robo3* | [58] |
|  | *Rtl1* | [24] |
|  | *Sema3e* | [59] |
|  | *Sim1* | [60,61] |
|  | *Slc6a1* | [10] |
|  | *Slc10a4* | [17] |
|  | *Slc26a7* | [10] |
|  | *Smoc1* | [3] |
|  | *Sp9* | [7,62] |
|  | *Sstr4* | [11] |
|  | *Stk33* | [63] |
|  | *Tcerg1l* | [17] |
|  | *Tesc* | [64] |
|  | *Th* | [65] |
|  | *Tmcc3* | [3] |
|  | *Tmem163* | [66] |
|  | *Vax1* | [5,7,67] |
|  | *Wif1* | [68,69] |
|  |  |  |
| **Extra-telencephalic** | *Fam222a* | [70] |
|  | *Foxi3* | [29] |
|  | *Gpd1* | [10] |
|  | *Hepacam2* | [3] |
|  | *Irx1* | [71] |
|  | *Kcns2* | [3] |
|  | *Kirrel2* | [72] |
|  | *Klhl35* | [3,73] |
|  | *Lgr6* | [74] |
|  | *Lhx3* | [75] |
|  | *Msx3* | [76] |
|  | *Nphs1* | [77] |
|  | *Olig3* | [78,79] |
|  | *Otol1* | [80] |
|  | *Pax7* | [81] |
|  | *Pgr* | [3,73] |
|  | *Prdm13* | [82,83] |
|  | *Ptf1a* | [56,84] |
|  | *Sox14* | [85] |
|  | *Stat4* | [86] |
|  | *Trp63* | [73] |
|  | *Vsx2* | [87] |

**References**

1. Sandberg M, Flandin P, Silberberg S, Su-Feher L, Price JD, Hu JS, et al. Transcriptional Networks Controlled by NKX2-1 in the Development of Forebrain GABAergic Neurons. Neuron. 2016 Sep 21;91(6):1260-1275.

2. Anthony TE, Heintz N. The folate metabolic enzyme ALDH1L1 is restricted to the midline of the early CNS, suggesting a role in human neural tube defects. J Comp Neurol. 2007 Jan 10;500(2):368-83.

3. <https://gp3.mpg.de/>

4. Gritli-Linde A, Vaziri Sani F, Rock JR, Hallberg K, Iribarne D, Harfe BD, et al. Expression patterns of the Tmem16 gene family during cephalic development in the mouse. Gene Expr Patterns. 2009 Mar;9(3):178-91.

5. Long JE, Swan C, Liang WS, Cobos I, Potter GB, Rubenstein JL. Dlx1&2 and Mash1 transcription factors control striatal patterning and differentiation through parallel and overlapping pathways. J Comp Neurol. 2009 Feb 1;512(4):556-72.

6. Oishi K, Watatani K, Itoh Y, Okano H, Guillemot F, Nakajima K, et al. Selective induction of neocortical GABAergic neurons by the PDK1-Akt pathway through activation of Mash1. Proc Natl Acad Sci U S A. 2009 Aug 4;106(31):13064-9.

7. Wang B, Long JE, Flandin P, Pla R, Waclaw RR, Campbell K, et al. Loss of Gsx1 and Gsx2 function rescues distinct phenotypes in Dlx1/2 mutants. J Comp Neurol. 2013 May 1;521(7):1561-84.

8. Mo Z, Li S, Yang X, Xiang M. Role of the Barhl2 homeobox gene in the specification of glycinergic amacrine cells. Development. 2004 Apr;131(7):1607-18.

9. Ding Q, Joshi PS, Xie ZH, Xiang M, Gan L. BARHL2 transcription factor regulates the ipsilateral/contralateral subtype divergence in postmitotic dI1 neurons of the developing spinal cord. Proc Natl Acad Sci U S A. 2012 Jan 31;109(5):1566-71.

10. Visel A, Thaller C, Eichele G. GenePaint.org: an atlas of gene expression patterns in the mouse embryo. Nucleic Acids Res. 2004 Jan 1;32(Database issue):D552-6.

11. Magdaleno S, Jensen P, Brumwell CL, Seal A, Lehman K, Asbury A, et al. BGEM: an in situ hybridization database of gene expression in the embryonic and adult mouse nervous system. PLoS Biol. 2006 Apr;4(4):e86.

12. Monteiro CB, Midão L, Rebelo S, Reguenga C, Lima D, Monteiro FA. Zinc finger transcription factor Casz1 expression is regulated by homeodomain transcription factor Prrxl1 in embryonic spinal dorsal horn late-born excitatory interneurons. Eur J Neurosci. 2016 Jun;43(11):1449-59.

13. Vacalla CM, Theil T. Cst, a novel mouse gene related to Drosophila Castor, exhibits dynamic expression patterns during neurogenesis and heart development. Mech Dev. 2002 Oct;118(1-2):265-8.

14. Kitajima K, Koshimizu U, Nakamura T. Expression of a novel type of classic cadherin, PB-cadherin in developing brain and limb buds. Dev Dyn. 1999 Jul;215(3):206-14.

15. Mayer M, Bercsényi K, Géczi K, Szabó G, Lele Z. Expression of two type II cadherins, Cdh12 and Cdh22 in the developing and adult mouse brain. Gene Expr Patterns. 2010 Oct-Dec;10(7-8):351-60.

16. Libé-Philippot B, Michel V, Boutet de Monvel J, Le Gal S, Dupont T, Avan P, et al. Auditory cortex interneuron development requires cadherins operating hair-cell mechanoelectrical transduction. Proc Natl Acad Sci U S A. 2017 Jul 25;114(30):7765-7774.

17. Diez-Roux G, Banfi S, Sultan M, Geffers L, Anand S, Rozado D, et al. A high-resolution anatomical atlas of the transcriptome in the mouse embryo. PLoS Biol. 2011 Jan 18;9(1):e1000582.

18. Aracri P, Meneghini S, Coatti A, Amadeo A, Becchetti A. α4β2∗ nicotinic receptors stimulate GABA release onto fast-spiking cells in layer V of mouse prefrontal (Fr2) cortex. Neuroscience. 2017 Jan 6;340:48-61.

19. Lischinsky JE, Sokolowski K, Li P, Esumi S, Kamal Y, Goodrich M, et al. Embryonic transcription factor expression in mice predicts medial amygdala neuronal identity and sex-specific responses to innate behavioral cues. Elife. 2017 Mar13;6:e21012.

20. Shimogori T, Lee DA, Miranda-Angulo A, Yang Y, Wang H, Jiang L, et al. A genomic atlas of mouse hypothalamic development. Nat Neurosci. 2010 Jun;13(6):767-75.

21. Romand R, Albuisson E, Niederreither K, Fraulob V, Chambon P, Dollé P. Specific expression of the retinoic acid-synthesizing enzyme RALDH2 during mouse inner ear development. Mech Dev. 2001 Aug;106(1-2):185-9.

22. Campos-Barros A, Amma LL, Faris JS, Shailam R, Kelley MW, Forrest D. Type 2 iodothyronine deiodinase expression in the cochlea before the onset of hearing. Proc Natl Acad Sci U S A. 2000 Feb 1;97(3):1287-92.

23. Bauer M, Szulc J, Meyer M, Jensen CH, Terki TA, Meixner A, et al. Delta-like 1 participates in the specification of ventral midbrain progenitor derived dopaminergic neurons. J Neurochem. 2008 Feb;104(4):1101-15.

24. Seibt J, Armant O, Le Digarcher A, Castro D, Ramesh V, Journot L, et al. Expression at the imprinted dlk1-gtl2 locus is regulated by proneural genes in the developing telencephalon. PLoS One. 2012;7(11):e48675.

25. Kohtz JD, Fishell G. Developmental regulation of EVF-1, a novel non-coding RNA transcribed upstream of the mouse Dlx6 gene. Gene Expr Patterns. 2004 Jul;4(4):407-12.

26. Hermans-Borgmeyer I, Süsens U, Borgmeyer U. Developmental expression of the estrogen receptor-related receptor gamma in the nervous system during mouse embryogenesis. Mech Dev. 2000 Oct;97(1-2):197-9.

27. Yabut OR, Ng HX, Yoon K, Arela JC, Ngo T, Pleasure SJ. The Neocortical Progenitor Specification Program Is Established through Combined Modulation of SHH and FGF Signaling. J Neurosci. 2020 Sep 2;40(36):6872-6887.

28. Le TN, Zhou QP, Cobos I, Zhang S, Zagozewski J, Japoni S, et al. GABAergic Interneuron Differentiation in the Basal Forebrain Is Mediated through Direct Regulation of Glutamic Acid Decarboxylase Isoforms by Dlx Homeobox Transcription Factors. J Neurosci. 2017 Sep 6;37(36):8816-8829.

29. Urness LD, Paxton CN, Wang X, Schoenwolf GC, Mansour SL. FGF signaling regulates otic placode induction and refinement by controlling both ectodermal target genes and hindbrain Wnt8a. Dev Biol. 2010 Apr 15;340(2):595-604.

30. Hevner RF, Miyashita-Lin E, Rubenstein JL. Cortical and thalamic axon pathfinding defects in Tbr1, Gbx2, and Pax6 mutant mice: evidence that cortical and thalamic axons interact and guide each other. J Comp Neurol. 2002 May 20;447(1):8-17.

31. Kohli V, Nardini D, Ehrman LA, Waclaw RR. Characterization of Glcci1 expression in a subpopulation of lateral ganglionic eminence progenitors in the mouse telencephalon. Dev Dyn. 2018 Jan;247(1):222-228.

32. Szucsik JC, Witte DP, Li H, Pixley SK, Small KM, Potter SS. Altered forebrain and hindbrain development in mice mutant for the Gsh-2 homeobox gene. Dev Biol. 1997 Nov 15;191(2):230-42.

33. Corbin JG, Rutlin M, Gaiano N, Fishell G. Combinatorial function of the homeodomain proteins Nkx2.1 and Gsh2 in ventral telencephalic patterning. Development. 2003 Oct;130(20):4895-906.

34. Corbin JG, Gaiano N, Machold RP, Langston A, Fishell G. The Gsh2 homeodomain gene controls multiple aspects of telencephalic development. Development. 2000 Dec;127(23):5007-20.

35. Rallu M, Machold R, Gaiano N, Corbin JG, McMahon AP, Fishell G. Dorsoventral patterning is established in the telencephalon of mutants lacking both Gli3 and Hedgehog signaling. Development. 2002 Nov;129(21):4963-74.

36.Toresson H, Potter SS, Campbell K. Genetic control of dorsal-ventral identity in the telencephalon: opposing roles for Pax6 and Gsh2. Development. 2000 Oct;127(20):4361-71.

37. Yun K, Potter S, Rubenstein JL. Gsh2 and Pax6 play complementary roles in dorsoventral patterning of the mammalian telencephalon. Development. 2001 Jan;128(2):193-205.

38. Song H, Lee B, Pyun D, Guimera J, Son Y, Yoon J, et al. Ascl1 and Helt act combinatorially to specify thalamic neuronal identity by repressing Dlxs activation. Dev Biol. 2015 Feb 15;398(2):280-91.

39. Miyoshi G, Bessho Y, Yamada S, Kageyama R. Identification of a novel basic helix-loop-helix gene, Heslike, and its role in GABAergic neurogenesis. J Neurosci. 2004 Apr 7;24(14):3672-82.

40. Moreno E, Hoffmann H, Gonzalez-Sepúlveda M, Navarro G, Casadó V, Cortés A, et al. Dopamine D1-histamine H3 receptor heteromers provide a selective link to MAPK signaling in GABAergic neurons of the direct striatal pathway. J Biol Chem. 2011 Feb 18;286(7):5846-54.

41. Kanazawa K, Imaizumi K, Mori T, Honma Y, Tojo M, Tanno Y, et al. Expression pattern of a novel death-promoting gene, DP5, in the developing murine nervous system. Brain Res Mol Brain Res. 1998 Mar 1;54(2):316-20.

42. Frazer S, Prados J, Niquille M, Cadilhac C, Markopoulos F, Gomez L, et al. Transcriptomic and anatomic parcellation of 5-HT3AR expressing cortical interneuron subtypes revealed by single-cell RNA sequencing. Nat Commun. 2017 Jan 30;8:14219.

43. Pfaff SL, Mendelsohn M, Stewart CL, Edlund T, Jessell TM. Requirement for LIM homeobox gene Isl1 in motor neuron generation reveals a motor neuron-dependent step in interneuron differentiation. Cell. 1996 Jan 26;84(2):309-20.

44. Elshatory Y, Gan L. The LIM-homeobox gene Islet-1 is required for the development of restricted forebrain cholinergic neurons. J Neurosci. 2008 Mar 26;28(13):3291-7.

45. Wyeth MS, Pelkey KA, Yuan X, Vargish G, Johnston AD, Hunt S, et al. Neto Auxiliary Subunits Regulate Interneuron Somatodendritic and Presynaptic Kainate Receptors to Control Network Inhibition. Cell Rep. 2017 Aug 29;20(9):2156-2168.

46. Sussel L, Marin O, Kimura S, Rubenstein JL. Loss of Nkx2.1 homeobox gene function results in a ventral to dorsal molecular respecification within the basal telencephalon: evidence for a transformation of the pallidum into the striatum. Development. 1999 Aug;126(15):3359-70.

47. Inaki K, Nishimura S, Nakashiba T, Itohara S, Yoshihara Y. Laminar organization of the developing lateral olfactory tract revealed by differential expression of cell recognition molecules. J Comp Neurol. 2004 Nov 15;479(3):243-56.

48. Sato Y, Suzuki S, Iijima Y, Iijima T. Neuroligin-induced presynaptic differentiation through SLM2-mediated splicing modifications of neurexin in cerebellar cultures. Biochem Biophys Res Commun. 2017 Nov 18;493(2):1030-1036.

49. Püschel AW, Betz H. Neurexins are differentially expressed in the embryonic nervous system of mice. J Neurosci. 1995 Apr;15(4):2849-56.

50. PuschelPüschel AW. Divergent properties of mouse netrins. Mech Dev. 1999 May;83(1-2):65-75.

51. Salminen M, Meyer BI, Bober E, Gruss P. Netrin 1 is required for semicircular canal formation in the mouse inner ear. Development. 2000 Jan;127(1):13-22.

52. Jamuar SS, Schmitz-Abe K, D'Gama AM, Drottar M, Chan WM, Peeva M, et al. Biallelic mutations in human DCC cause developmental split-brain syndrome. Nat Genet. 2017 Apr;49(4):606-612.

53. Silbereis JC, Nobuta H, Tsai HH, Heine VM, McKinsey GL, Meijer DH, et al. Olig1 function is required to repress dlx1/2 and interneuron production in Mammalian brain. Neuron. 2014 Feb 5;81(3):574-87,

54. Miyoshi G, Butt SJ, Takebayashi H, Fishell G. Physiologically distinct temporal cohorts of cortical interneurons arise from telencephalic Olig2-expressing precursors. J Neurosci. 2007 Jul 18;27(29):7786-98.

55. <https://developingmouse.brain-map.org/>

56. Bröhl D, Strehle M, Wende H, Hori K, Bormuth I, Nave KA, et al. A transcriptional network coordinately determines transmitter and peptidergic fate in the dorsal spinal cord. Dev Biol. 2008 Oct 15;322(2):381-93.

57. Shimazaki T, Arsenijevic Y, Ryan AK, Rosenfeld MG, Weiss S. A role for the POU-III transcription factor Brn-4 in the regulation of striatal neuron precursor differentiation. EMBO J. 1999 Jan 15;18(2):444-56.

58. Barber M, Di Meglio T, Andrews WD, Hernández-Miranda LR, Murakami F, Chédotal A, et al. The role of Robo3 in the development of cortical interneurons. Cereb Cortex. 2009 Jul;19 Suppl 1(Suppl 1):i22-31.

59. Bribián A, Nocentini S, Llorens F, Gil V, Mire E, Reginensi D, et al. Sema3E/PlexinD1 regulates the migration of hem-derived Cajal-Retzius cells in developing cerebral cortex. Nat Commun. 2014 Jun 27;5:4265.

60. Blacklaws J, Deska-Gauthier D, Jones CT, Petracca YL, Liu M, Zhang H, et al. Sim1 is required for the migration and axonal projections of V3 interneurons in the developing mouse spinal cord. Dev Neurobiol. 2015 Sep;75(9):1003-17.

61. Fan CM, Kuwana E, Bulfone A, Fletcher CF, Copeland NG, Jenkins NA, et al. Expression patterns of two murine homologs of Drosophila single-minded suggest possible roles in embryonic patterning and in the pathogenesis of Down syndrome. Mol Cell Neurosci. 1996 Jan;7(1):1-16.

62. Li J, Wang C, Zhang Z, Wen Y, An L, Liang Q, et al. Transcription Factors Sp8 and Sp9 Coordinately Regulate Olfactory Bulb Interneuron Development. Cereb Cortex. 2018 Sep 1;28(9):3278-3294.

63. Castro DS, Skowronska-Krawczyk D, Armant O, Donaldson IJ, Parras C, Hunt C, et al. Proneural bHLH and Brn proteins coregulate a neurogenic program through cooperative binding to a conserved DNA motif. Dev Cell. 2006 Dec;11(6):831-44.

64. Bao Y, Hudson QJ, Perera EM, Akan L, Tobet SA, Smith CA, et al. Expression and evolutionary conservation of the tescalcin gene during development. Gene Expr Patterns. 2009 Jun;9(5):273-81.

65. Wang M, Cai E, Fujiwara N, Fones L, Brown E, Yanagawa Y, et al. Odorant Sensory Input Modulates DNA Secondary Structure Formation and Heterogeneous Ribonucleoprotein Recruitment on the Tyrosine Hydroxylase and Glutamic Acid Decarboxylase 1 Promoters in the Olfactory Bulb. J Neurosci. 2017 May 3;37(18):4778-4789.

66. Hoerder-Suabedissen A, Wang WZ, Lee S, Davies KE, Goffinet AM, Rakić S, et al. Novel markers reveal subpopulations of subplate neurons in the murine cerebral cortex. Cereb Cortex. 2009 Aug;19(8):1738-50.

67. Hallonet M, Hollemann T, Wehr R, Jenkins NA, Copeland NG, Pieler T, et al. Vax1 is a novel homeobox-containing gene expressed in the developing anterior ventral forebrain. Development. 1998 Jul;125(14):2599-610.

68. Geng R, Noda T, Mulvaney JF, Lin VY, Edge AS, Dabdoub A. Comprehensive Expression of Wnt Signaling Pathway Genes during Development and Maturation of the Mouse Cochlea. PLoS One. 2016 Feb 9;11(2):e0148339.

69. Hu YA, Gu X, Liu J, Yang Y, Yan Y, Zhao C. Expression pattern of Wnt inhibitor factor 1(Wif1) during the development in mouse CNS. Gene Expr Patterns. 2008 Sep;8(7-8):515-22.

70. Nakano Y, Jahan I, Bonde G, Sun X, Hildebrand MS, Engelhardt JF, et al. A mutation in the Srrm4 gene causes alternative splicing defects and deafness in the Bronx waltzer mouse. PLoS Genet. 2012;8(10):e1002966.

71. Bosse A, Zülch A, Becker MB, Torres M, Gómez-Skarmeta JL, Modolell J, et al. Identification of the vertebrate Iroquois homeobox gene family with overlapping expression during early development of the nervous system. Mech Dev. 1997 Dec;69(1-2):169-81.

72. Sun C, Kilburn D, Lukashin A, Crowell T, Gardner H, Brundiers R, et al. Kirrel2, a novel immunoglobulin superfamily gene expressed primarily in beta cells of the pancreatic islets. Genomics. 2003 Aug;82(2):130-42.

73. <http://www.informatics.jax.org/gxd>

74. Zhang Y, Chen Y, Ni W, Guo L, Lu X, Liu L, et al. Dynamic expression of Lgr6 in the developing and mature mouse cochlea. Front Cell Neurosci. 2015 May 12;9:165.

75. Thaler JP, Lee SK, Jurata LW, Gill GN, Pfaff SL. LIM factor Lhx3 contributes to the specification of motor neuron and interneuron identity through cell-type-specific protein-protein interactions. Cell. 2002 Jul 26;110(2):237-49.

76. Liu Y, Helms AW, Johnson JE. Distinct activities of Msx1 and Msx3 in dorsal neural tube development. Development. 2004 Mar;131(5):1017-28.

77. Nishida K, Hoshino M, Kawaguchi Y, Murakami F. Ptf1a directly controls expression of immunoglobulin superfamily molecules Nephrin and Neph3 in the developing central nervous system. J Biol Chem. 2010 Jan 1;285(1):373-80.

78. Zechner D, Müller T, Wende H, Walther I, Taketo MM, Crenshaw EB 3rd, et al. Bmp and Wnt/beta-catenin signals control expression of the transcription factor Olig3 and the specification of spinal cord neurons. Dev Biol. 2007 Mar 1;303(1):181-90.

79. Müller T, Anlag K, Wildner H, Britsch S, Treier M, Birchmeier C. The bHLH factor Olig3 coordinates the specification of dorsal neurons in the spinal cord. Genes Dev. 2005 Mar 15;19(6):733-43.

80. Xu Y, Zhang Y, Lundberg YW. Spatiotemporal differences in otoconial gene expression. Genesis. 2016 Dec;54(12):613-625.

81. Burrill JD, Moran L, Goulding MD, Saueressig H. PAX2 is expressed in multiple spinal cord interneurons, including a population of EN1+ interneurons that require PAX6 for their development. Development. 1997 Nov;124(22):4493-503.

82. Mona B, Uruena A, Kollipara RK, Ma Z, Borromeo MD, Chang JC, et al. Repression by PRDM13 is critical for generating precision in neuronal identity. Elife. 2017 Aug 29;6:e25787.

83. Chang JC, Meredith DM, Mayer PR, Borromeo MD, Lai HC, Ou YH, et al. Prdm13 mediates the balance of inhibitory and excitatory neurons in somatosensory circuits. Dev Cell. 2013 Apr 29;25(2):182-95.

84. Huang M, Huang T, Xiang Y, Xie Z, Chen Y, Yan R, et al. Ptf1a, Lbx1 and Pax2 coordinate glycinergic and peptidergic transmitter phenotypes in dorsal spinal inhibitory neurons. Dev Biol. 2008 Oct 15;322(2):394-405.

85. Hargrave M, Karunaratne A, Cox L, Wood S, Koopman P, Yamada T. The HMG box transcription factor gene Sox14 marks a novel subset of ventral interneurons and is regulated by sonic hedgehog. Dev Biol. 2000 Mar 1;219(1):142-53.

86. Gray PA, Fu H, Luo P, Zhao Q, Yu J, Ferrari A, et al. Mouse brain organization revealed through direct genome-scale TF expression analysis. Science. 2004 Dec 24;306(5705):2255-7.

87. Liu IS, Chen JD, Ploder L, Vidgen D, van der Kooy D, Kalnins VI, et al. Developmental expression of a novel murine homeobox gene (Chx10): evidence for roles in determination of the neuroretina and inner nuclear layer. Neuron. 1994 Aug;13(2):377-93.
