# Supplementary material for: Pax6 limits the competence of developing cerebral cortical cells to respond to inductive intercellular signals
Source: PLoS Biol. 2022 Sep 6;20(9):e3001563. doi: 10.1371/journal.pbio.3001563 (PMC9481180; doi:10.1371/journal.pbio.3001563)
Supplement: Attachment — Submitted filename: Response to Reviewers.docx [file pbio.3001563.s034.docx]

**Response to Reviewer 1**

*The manuscript by Manuel et al. titled "Pax6 limits the competence of developing cerebral cortical cells" systematically dissects the role of Pax6 in corticogenesis by using a conditional knockout mouse model. The authors conclude that Pax6 expression is not necessary for the formation of excitatory neurons but rather acts as a protective factor to block extrinsic signals leading to altered cell fates. This work is quite extensive and reaffirms many results from prior studies thus impacting the overall novelty. However, with addition of the single cell data, this work can be an important modern resource for those studying corticogenesis.*

1. *In the transcriptomic analysis, it is unclear the decision to choosing the thresholds for the log fold changes used to call differential gene expression. For example, the LFC > 0 in Figure 1B vs LFC >= 1 in Figure 1E.*

**In the revision, we have moved Fig. 1 to become Supplementary Fig. 2 (Fig. S2), to help make the first part of the paper more concise, as suggested by Reviewer 2. We have added a statement to the legend of Fig. S2 panel D to make clear that the only threshold applied here was an arbitrary one commonly used to create a set of genes that at least doubled or halved their expression levels. This was applied only in the final stages of this analysis, to give us a dataset of manageable size from which to gain a sense of the types, normal expression patterns and possible functions of the genes most affected by Pax6 deletion. By restricting this exercise to the genes showing the largest changes, it became feasible to annotate them manually ourselves (e.g. Table S3) as well as using existing bioinformatic tools (e.g. Table S2). The insights gained helped guide our thinking in the early stages of the project. A full list of all significant gene expression changes with no threshold applied is given in Table S1.**

2. *It is unclear in the text/methods how the aRGP population was determined as a different cell type from RGPs in the UMAP (Figure 2B). It may be helpful to more broadly define the characteristics of aRGPs compared to RGPs.*

**The population that we labelled as aRGP emerged when we applied a clustering algorithm to the dataset at the resolution required to separate the other major known cell types (new Fig. 1B). We discuss some of the major differences that caused the algorithm to separate aRGPs from RGPs (these data can now be found in new Fig. S3) and Table S4 gives an extensive list of genes showing differences in average levels of expression between aRGPs and RGPs. Opinions will likely differ on whether it is helpful to consider aRGPs “*a different cell type from RGPs*”. We prefer to consider aRGPs as variants of RGPs whose production is greatly enhanced by Pax6 removal.**

3. *In Figure 4, the RNA velocity analysis shows that RGPs and IP cells move toward deep and superficial layer neuron cell types, but it is a bit confusing that the directionality continues from superficial layer neurons to deep layer identity.*

**RNA Velocity is a method used on data from single cell RNA sequencing to infer the dynamical state changes of developing cells. In our Velocity plots, the direction and size of each arrow indicates the direction and speed of movement of each cell towards its predicted future state. As the reviewer says, arrows in clusters of cells identified as newly generated superficial layer neurons point in the direction of clusters identified as deep layer neurons. And yet, as we know from countless studies, superficial layer neurons do not progress to become deep layer neurons. How can this apparent conundrum be resolved?**

**The answer is that, at this young age, the arrows of cells in more superficial layers point to states that do not yet exist. The directionality in these parts of the Velocity plots is from less mature to more mature neurons (this progression is seen in the RNA Velocity inferences of others examining embryonic cortical development with single cell RNA sequencing, e.g. Noack et al., 2022, *Nature Neuroscience* 25; 154-167). At E14.5, layer 6 neurons are the most mature. Many layer 6 cells (towards which other layer 6 cells are pointing) lack arrows, indicating that they have achieved a relatively mature state (bottom right of the plots now shown in Fig. 2). In the clusters of more superficial layer neurons, there is some evidence of relatively mature neurons with no - or very small - arrows in regions that will probably become the locations of more mature layer 2-5 neurons as development proceeds. Essentially, because the layer-specific identities of neurons are still maturing, the Velocity arrows of cells in each layer are pointing to where their more mature states would be represented in plots generated at later ages, which is in the same direction as the more mature neurons of the deep layers.**

4. *In the embryonic data, it would help to validate some of the aRGP cells within the developing cortex, like Fos or Meg3.*

**We carried out a new experiment in which we immunostained control and *Pax6* cKO E13.5 cortex for Fos expression. The results indicated increased levels of Fos in *Pax6* cKO cortex, as would be predicted from the single cell RNAseq data. This is now included in Fig. S3 and referred to in the text (line 176).**

5. *The interpretation of the Bmp treatment in Pax6 cKO cortices suggest that Bmp4 can suppress the ventral marker Gsx2. Is there any data that can distinguish between suppression versus simply the differentiation of Gsx2+ cells?*

**We measured the effects of Bmp treatment on *Gsx2* mRNA levels and numbers of Gsx2+ cells. The latter measurements suggested that fewer Gsx2+ cells were produced as Bmp levels were increased (shown in new Fig. 9C). This was not explained as clearly as it should have been in the original text and we have modified lines 605-606 and 628-629 accordingly.**

**Response to Reviewer 2**

*In this manuscript, Manuel et al. describe the role of Pax6 development of the neocortex. They analyzed transcriptome changes in different cell types of Pax6 mutant cortex by single cell RNA Seq as well as by in situ hybridization and immunohistochemistry.*

*They also carried out in vitro experiments aiming to test responsiveness of Pax6 deficient cells to developmental morphogens, such as Shh and BMP.*

*The main conclusion of the manuscript is that Pax6 controls competence of cortical cells to environmental cues.*

*Essentially the manuscript can be divided into two parts: the first part describes Pax6 mutant phenotype in the developing neocortex, the second part investigates the role of in vitro and in vivo manipulations of neocortical tissue and cells in the presence of activators/inhibitors of SHH and BMP signaling pathways. In the first part, Figures 1-9 present very comprehensive and thorough analysis of molecular changes at single cell type level in the Pax6 mutant neocortex…I think that this part can be substantially reduced and half of the figures can go to the supplement. I think the authors should make this part more focused on eGCs cells in Pax6 mutant. Another aspect that is important for the main message of the manuscript, is presence of glutamatergic neurons in Pax6 mutants. All the data that confirm or repeat published observations, like "ventralization" of the dorsal telencephalon, proliferation characteristics of Pax6 deficient cells etc. do not have to be shown and discussed so extensively. Instead I suggest to focus on the main message of this part that is important to understand the second part of the manuscript…My recommendation: to shorten the manuscript substantially, with more focus on novel findings and reduce the number of figures to seven-eight.*

**We have streamlined the presentation in line with these suggestions. There are now nine figures in total. Specifically, we moved former Fig. 1 (bulk RNAseq) to Supplementary Figures. We reduced the presentation of single cell RNAseq data from three figures to two by moving some aspects of the analysis to Supplementary Figures. We reduced the presentation of changes in tissue sections from two figures to one by moving many images of stained sections to Supplementary Figures and retaining the quantifications, which summarize findings important for the second part of the paper. We have moved the images of gene expression loss to Supplementary Figures.**
